# Replication-associated mechanisms contribute to an increased CpG > TpG mutation burden in mismatch repair-deficient cancers

**DOI:** 10.1186/s13073-025-01525-6

**Published:** 2025-08-25

**Authors:** Joseph C. Ward, Ignacio Soriano, Steve Thorn, Juan Fernández-Tajes, Kitty Sherwood, Güler Gül, Joost Scheffers, Anna Frangou, Ben Kinnersley, Ioannis Kafetzopoulos, Duncan Sproul, Sara Galavotti, Claire Palles, Andreas J. Gruber, David N. Church, Ian Tomlinson

**Affiliations:** 1 Department of Oncology, University of Oxford, Old Road Campus Research Building, Roosevelt Drive, Oxford, OX3 7DQ UK; 2 Cancer Research UK Scotland Centre, Institute of Genetics and Cancer, University of Edinburgh, Western General Hospital, Crewe Road, Edinburgh, EH4 2XU UK; 3 Cancer Immunogenomics, Leiden University Medical Center, Leiden, Netherlands; 4 Big Data Institute, University of Oxford, Li Ka Shing Centre for Health Information and Discovery, Oxford, OX3 7LF UK; 5 Max Planck Institute for Molecular Cell Biology and Genetics, Dresden, Germany; 6 Department of Oncology, UCL Cancer Institute, University College London, Paul O’Gorman Building, Huntley Street, London, WC1E 6DD UK; 7 MRC Human Genetics Unit, Institute of Genetics and Cancer, University of Edinburgh, Western General Hospital, Crewe Road, Edinburgh, EH4 2XU UK; 8 Institute of Cancer and Genomic Sciences, University of Birmingham, Vincent Drive, Edgbaston, Birmingham, B15 2TT UK; 9 Department of Biology, University of Konstanz, Konstanz, Germany; 10 Manchester Cancer Research Centre, Division of Cancer Sciences, University of Manchester, Wilmslow Road, Manchester, M20 4GJ UK; 11 Centre for Human Genetics, University of Oxford, Roosevelt Drive, Oxford, OX3 7BN UK; 12 Oxford NIHR Comprehensive Biomedical Research Centre, Oxford University Hospitals NHS Foundation Trust, John Radcliffe Hospital, Headley Way, Oxford, OX3 9DU UK

**Keywords:** Mutation signatures, Colorectal cancer, Mismatch repair deficiency, DNA repair, Mutagenesis

## Abstract

**Background:**

Single base substitution (SBS) mutations, particularly C > T and T > C, are increased owing to unrepaired DNA replication errors in mismatch repair-deficient (MMRd) cancers. Excess CpG > TpG mutations have been reported in MMRd cancers defective in mismatch detection (dMutSα), but not in mismatch correction (dMutLα). Somatic CpG > TpG mutations conventionally result from unrepaired spontaneous deamination of 5’-methylcytosine throughout the cell cycle, causing T:G mismatches and signature SBS1. It has been proposed that MutSα detects those mismatches, prior to error correction by base excision repair (BER). However, other evidence appears inconsistent with that hypothesis: for example, MutSα is specifically expressed in S/G_2_ phases of the cell cycle, and defects in replicative DNA polymerase proofreading specifically cause excess CpG > TpG mutations in signature SBS10b.

**Methods:**

We analysed mutation spectra and COSMIC mutation signatures in whole-genome sequencing data from 1803 colorectal cancers (164 dMutLα, 20 dMutSα) and 596 endometrial cancers (103 dMutLα, 9 dMutSα) from the UK 100,000 Genomes Project. We mapped each C > T mutation to its genomic features, including normal DNA methylation state, replication timing, transcription strand, and replication strand, to investigate the mechanism(s) by which these mutations arise.

**Results:**

We confirmed that dMutSα tumours specifically had higher CpG > TpG burdens than dMutLα tumours. We could fully reconstitute the observed dMutSα CpG > TpG mutation spectrum by adding CpG > TpG mutations in proportion to their SBS1 activity to the dMutLα spectrum. However, other evidence indicated that the SBS1 excess in dMutSα cancers did not come from 5’-methylcytosine deamination alone: non-CpG C > T mutations were also increased in dMutSα cancers; and, in contrast to tumours deficient in BER, CpG > TpG mutations were biased to the leading DNA replication strand, at similar levels in dMutSα and dMutLα cancers, suggesting an origin in DNA replication. Other substitution mutations usually corrected by BER were not increased in dMutSα tumours.

**Conclusions:**

There is a CpG > TpG and SBS1 excess specific to dMutSα MMRd tumours, consistent with previous reports, and we find a general increase in somatic C > T mutations. Contrary to some other studies, the similar leading replication strand bias in both dMutSα and dMutLα tumours indicates that at least some of the excess CpG > TpG mutations arise via DNA replication errors, and not primarily via the replication-independent deamination of 5’-methylcytosine.

**Supplementary Information:**

The online version contains supplementary material available at 10.1186/s13073-025-01525-6.

## Background

Cytosine to thymine (C > T) mutations are probably the most common single-base substitution (SBS) mutations in normal tissues and tumours. The consensus view is that C > T changes at CpG sites (CpG > TpG) mostly arise from spontaneous deamination of 5’-methylcytosine (5’-MeC) to thymine or, less often, unmodified cytosine to uracil [1–4], occurring at any stage of the cell cycle. Many of the resulting T:G mismatches appear to be corrected by the base excision repair (BER) pathway, but some escape detection or are uncorrected, largely for unknown reasons. The Catalogue of Somatic Mutations in Cancer (COSMIC) mutation signature SBS1 (https://cancer.sanger.ac.uk/signatures/sbs/sbs1/) predominantly comprises NCG > NTG changes (where N is any base) [5] and the SBS1 burden generally accumulates with patient age [1, 2, 6]. We and others have shown that individuals with constitutive deficiency in the BER protein MBD4, which corrects T:G mismatches, develop tumours with a very high CpG > TpG mutation burden and a predominant mutational signature that is very similar to SBS1 [7–9].

Apart from SBS1, C > T mutations feature in several other SBS mutational signatures from human cancers and other sources [5]. Some of these signatures (for example, SBS5) contain many active mutational channels including C > T changes, while in other signatures, C > T changes comprise the major component. Examples of the latter (with their aetiology) include SBS2 (APOBEC), SBS6 (DNA mismatch repair deficiency, MMRd), SBS7a/b (UV light exposure), SBS10b (DNA polymerase ε proofreading deficiency), SBS15 (MMRd), SBS19 (unknown), SBS23 (unknown), SBS30 (NTHL1 deficiency), SBS31 (platinum therapy), SBS32 (azathioprine), SBS35 (platinum therapy), SBS42 (haloalkane exposure), SBS44 (MMRd), SBS84 (activation-induced cytidine deaminase), SBS87 (thiopurine treatment), and SBS97 (unknown).

A tendency toward C > T changes is present in most of the MMRd-specific SBS signatures, the two main exceptions being SBS21 and SBS26, that are typified by T > C base substitutions [5]. A number of doublet-base substitution (DBS) and insertion-deletion (ID) mutation signatures are also attributed to MMRd [5]. These include DBS7 (TT > NN), DBS10 (GC > TA) and ID7 (single base-pair deletions) [5], emphasising the different mutation profiles of MMRd cancers compared with their mismatch repair-proficient (MMRp) counterparts.

MMRd is a feature of several cancer types, most commonly carcinomas of the colorectum (CRC) and endometrium (EC) [10, 11]. Cancers usually acquire MMRd through defects in the MutSα mismatch recognition complex (usually *MSH2* or *MSH6* mutations) or the MutLα mismatch correction complex (mostly MLH1 promoter hypermethylation and transcriptional silencing, or occasionally *MLH1* or *PMS2* mutations). This results in an increased frequency of replication slippage mutations at short repeats (“microsatellite instability”, MSI) which typifies MMRd tumours, but the frequencies of multiple SBS, DBS, and ID mutations are also increased, as evidenced in the MMRd-specific mutational signatures described above.

MMR is classically regarded as a process of repair that is coupled to DNA replication, acting as a second string to replicative DNA polymerase proofreading [12, 13]. However, it has been proposed by Fang et al. [14] that SBS1 itself can be caused by MMR deficiency, who specifically found increased SBS1 in MMRd tumours caused by defects in the MutSα mismatch detection arm of MMR, rather than the MutLα component that initiates removal of the mismatched base. More controversially, it was proposed that MutSα plays a role outside DNA replication by detecting the T:G mismatches that result from spontaneous 5’-MeC deamination at any stage in the cell cycle [14]; once detected, these mismatches would usually be repaired by MBD4 and related enzymes [3, 15]. Thus, defects in the MutSα complex would lead to a generally increased burden of somatic CpG > TpG mutations, in addition to increased replication errors.

Here, we describe the somatic C > T mutational landscape from whole-genome sequencing (WGS) of a set of 1803 CRCs, including 310 MMRd tumours. We determine the frequencies of different C > T mutations according to their flanking bases in MutSα- and MutLα-deficient tumours (which we term dMutSα and dMutLα respectively). We also identify the mutational processes active in those tumours, and the associations of C > T changes with genomic features, such as DNA methylation state and replication timing. We compare C > T mutations in CRCs with those in a set of 596 ECs. The results support a role for MutSα in preventing C > T changes, but we also provide evidence that unrepaired deamination of 5’-MeC is not the only underlying cause.

## Methods

### Whole-genome sequencing data

We obtained WGS data from the UK 100,000 Genomes Project (100kGP) [16]. A detailed description of patient recruitment and sample processing has been published previously [17]. Briefly, samples were collected as a part of the 100kGP cancer programme across thirteen Genomic Medicine Centres. All participants provided written informed consent and had both blood and tumour samples taken. Only samples that met the following criteria were used in downstream analysis: (i) sample was a fresh-frozen primary tumour; (ii) no DNA-damaging chemotherapy or radiotherapy had been administered before sampling; (iii) the sequencing library was prepared using PCR-free methods; and (iv) contamination by other patient samples was < 1%. A total of 1803 CRCs and 596 ECs was included in our cohorts. Quality control, sequencing read alignment (hg38), and variant calling of WGS data were performed according to the standard pipeline for the 100kGP dataset [16], with somatic mutations called using Strelka (V2.4.7) [18]. The average depth of tumour and germline samples sequenced as a part of the 100kGP was about 100 × and 33x, respectively. Mutations were included in further analyses if they had a “PASS” flag in all quality control filters of the variant call file.

The MMRd status of each cancer was determined using the Detecting Microsatellite Instability by Next-Generation Sequencing (mSINGS) pipeline [19]. Cancers were classified as MMRd if they were MSI + according to mSINGS and had a tumour mutation burden of > 10 somatic coding mutations per Mb. We identified some MMRd cancers with pathogenic *MSH2*, *MSH6*, or *MLH1* mutations in the germline, all of which were accompanied by second hits in the form of somatic mutation or copy number alteration in the tumour. Copy number data for ECs was obtained from Battenberg [20], while in CRCs this was obtained from a consensus of Battenberg and ASCAT [21]. Somatic mutations in the above mismatch repair genes were classed as either heterozygous or homozygous by comparing the observed numbers of reference and alternative reads with those expected based on the tumour purity (*χ*^2^ test, *P* < 0.05) [22]. MMRd cancers with apparently pathogenic mutations in *PMS2* were excluded due to its many highly homologous pseudogenes, making genuine pathogenic mutations difficult to identify [23]. Germline or somatic DNA polymerase ε (*POLE*) or δ (*POLD1*) exonuclease domain mutations (EDMs), resulting in proofreading deficiency and previously reported as pathogenic, were also identified [24–26]. Any MMRd cancer with a pathogenic *POLE* or *POLD1* EDM was excluded from downstream analysis.

**Fig. 1:**
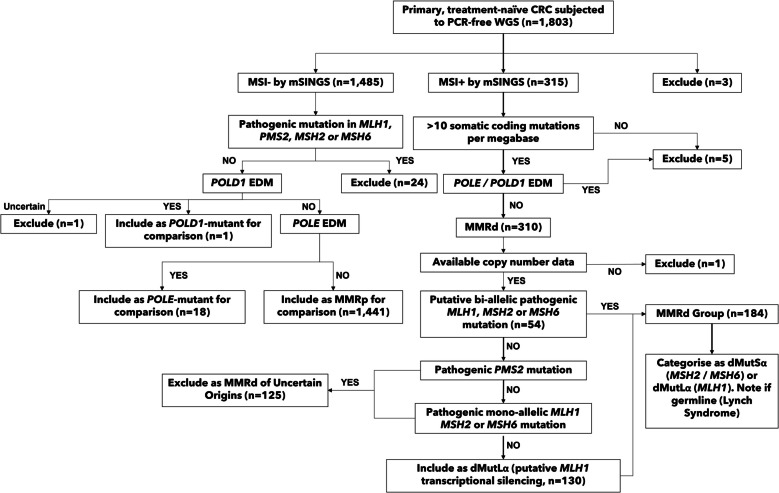
Colorectal cancers included in mutation analysis. A summary of the primary, treatment-naïve colorectal cancers (CRCs) subjected to PCR-free whole-genome sequencing analysis from V18 of the UK 100,000 Genomes Project. mSINGS = Detecting MSI by Next-Generation Sequencing. MMRp = Mismatch repair-proficient. MMRd = Mismatch repair-deficient. POLE = DNA polymerase ε. POLD1 = DNA polymerase δ

DNA methylation and transcriptome analyses were not performed in the 100kGP project. We thus necessarily identified putative *MLH1*-methylated cancers by excluding other causes of MSI/MMRd. We reasoned that specificity (lack of false assignments to either the dMutSα or dMutLα sub-groups) was more important than sensitivity (true assignment of all dMutSα and dMutLα cancers). We were careful to perform a stringent classification, such that the samples included in the analysis either (i) had two pathogenic mutations in *MLH1* or *MSH2* or *MSH6*, or (ii) had no evidence of any pathogenic mismatch repair gene (epi)mutation, including copy number changes. The stringent dMutSα/dMutLα classification on the 310 MMRd *POLE-* and *POLD1-*wildtype CRCs comprised the following (see Fig. 1): (i) we required evidence of bi-allelic, pathogenic *MSH2*,* MSH6*, or *MLH1* mutations to class a tumour as dMutSα or dMutLα; (ii) we took account of the phenomenon of secondary indel mutations in hypermutable short repeats in MMR genes that created truncated variants (e.g. *MSH6* p.Ile245fs, p.Thr1085fs and p.Thr1102fs), and by excluding these mutations, we prevented *MLH1-*silenced tumours being misclassified as dMutSα; and (iii) we excluded sub-clonal somatic variants (based on alternative read counts being lower than expected, using nominal significance levels and correction for tumour purity). Of the 310 CRCs, we could not assign or exclude a mutational cause of MMRd with high-confidence in 126. These CRCs were denoted as having “MMRd of Uncertain Origins” and comprised tumours with mono-allelic somatic mutations in the MutSα genes (*n* = 85) or *MLH1* (*n* = 12), cancers with *PMS2* mutations (*n* = 28, see above), or a lack of copy number data (*n* = 1). The remaining tumours were further partioned into Lynch syndrome (germline pathogenic mismatch repair gene mutation with somatic second hit or copy number alteration, *n* = 23), somatic bi-allelic mismatch repair mutant (including copy number changes, *n* = 31) and no identifiable mismatch repair mutation (*n* = 130). The last group were assumed to result from somatic silencing of *MLH1* expression by promoter methylation, which is long established as the most common underlying cause of MMRd, being present in ~ 75% of sporadic MSI + CRCs in multiple studies [27, 28].

We tested our classification method on CRCs from The Cancer Genome Atlas (TCGA-COADREAD, [29]) as used by Fang et al. [14]. Twenty-eight of the thirty-five CRCs classified as dMutLα by Fang et al. were also classified as dMutLα using our method (Additional file 1: Table S1). Of the five classified by Fang et al. as dMutSα, we correctly classified one and the rest were classified as “MMRd of Uncertain Origins” by either our method or by Fang et al. (Additional file 1: Table S1). We note that, while there were differences in the CRCs assigned to the “MMRd of Uncertain Origins” group, there were no instances of dMutLα CRCs being mis-classified as dMutSα or vice versa using our method. We also additionally performed the study's major analyses in the sub-set of cancers with confirmed bi-allelic mutations in order to check that associations were not artefacts of incorrect assignments as dMutLα or dMutSα.

### Mutation signature extraction

Mutation signatures from the COSMIC database (V3.2) were extracted from the somatic SBS mutations of each cancer using the trinucleotide context of each mutation as input for SigProfilerExtractor [30]. For mutation signature extraction, the minimum number of de novo signatures was set to one and the maximum to ten, with 200 NMF replicates. Separate analyses were performed on the whole cancer cohort (CRC or EC) and MMRd cancers with a known cause. De novo mutation signature extraction was also performed on MMRd CRCs and ECs, setting the number of de novo signatures to two, thus mimicking the work of Fang et al. [14]. When extracting signatures from exome data, the parameters used were as above, with the exception of setting the “exome” flag to “True”.

### Measures of mutation

We assessed SBS mutations in three contexts: (i) mononucleotide (six single base channels – C > A, C > G, C > T, T > A, T > C, T > G), (ii) 96-channel trinucleotide (ACA > AAA and 95 others, where the underlined base is substituted); and (iii) 96-channel signatures (https://cancer.sanger.ac.uk/signatures/sbs/), each of which combines different levels of the 96 channels. In this manuscript, we often split C > T mutations into CpG > TpG (i.e. C > T at CpG dinucleotides) and non-CpG C > T changes. Similar measures were also used for doublet base substitutions (DBS) and small indels (ID). In turn, mutations in each channel or signature were assessed in three ways in a tumour or set of tumours: (a) burdens (absolute numbers of a specified mutation type); (b) proportion or proportional activities (burden relative to the total burden of the specified class of mutation, often used in identifying and describing mutation signatures); and (c) prevalence (presence of the specified mutation type at any level > 0% *versus* absence). Each measure co-varies, but has specific, overlapping strengths. For example, burdens are useful for identifying hypermutant tumours, activity is useful when correcting for background levels that could vary with generalised genomic instability or environmental exposure, and prevalence is useful for determining whether a specific signature (and underlying mutational process) is active within a cancer, often in relation to a specific cause.

### Identification of kataegis regions

Regions of kataegis in CRCs were identified using a combination of MAFtools [31] and SeqKat [32]. Somatic mutations within these regions were identified from variant call files using BEDtools (V2.30.0) [33].

### Mapping mutations to molecular features

Fractional DNA methylation data from whole-genome bisulphite sequencing of the normal human colon was obtained from the RoadMap Epigenomics Consortium and lifted from hg19 to hg38 using the University of California Santa Cruz (UCSC) LiftOver tool [34, 35]. This provided a quantitative estimate of DNA methylation for 27,093,801 CpG sites. DNA replication timing data were produced for the CRC cell line HCT116 using the Repli-Seq method described by Marchal et al. [36], where replication timing (T) values were calculated for 10-kb regions of the genome. Transcription strand data were obtained from GENCODE, assigning genes to the trx + or trx − strands [37, 38], where trx + and trx − genes are respectively transcribed on the “top” and “bottom” DNA strand (as assigned in human genome annotation). Replication strand data were obtained from the study by Haradhvala et al. [39, 40], who used six lymphoblastoid cell lines from mother-father-offspring trios of European and West-African heritage. From these data, 20-kb regions were classified as left-replicating or right-replicating according to the consensus of replication origins in the region [39]. Somatic C > T mutations from each cancer, including nominally C > T and G > A changes, were binned to their corresponding DNA methylation state, replication timing, transcription strand, and replication strand in the reference data described above using BEDtools. C > T mutations were assigned to the coding or template transcription strands according to the precedent set by Vӧhringer et al. [37]. Similarly, C > T mutations were assigned to the leading or lagging replication strand according to the convention set out in previous studies [14, 39, 41, 42]. The numbers of CpG > TpG mutations mapping to each DNA methylation or replication timing bin were normalised against the number of CpG sites found within the bin according to the following formula, adapted from Sanders et al. [43] (Mutation Rate = M_x_/(N_x_/1,000,000)) where M_x_ represents the number of CpG > TpG mutations in bin x and N_x_ is the number of CpG sites in bin x (Additional file 1: Table S2).

### Statistical analysis

Mutation frequencies of each SBS, DBS, and ID channel were measured as both absolute burdens and activities (proportion of the total mutation burden) in each cancer. Following mutation signature extraction, we used the number of mutations (SBS, DBS, or ID) assigned to a COSMIC reference signature to calculate the activity of that signature in each cancer. Differences between discrete groups of quantitative variables were assessed using Wilcoxon tests in the R package “ggpubr” (V0.6.0) [44], unless otherwise stated. To assess the relationship between DNA methylation or replication timing and CpG > TpG mutation, a non-parametric Jonckheere-Terpstra test for trend (JT test) was performed using the R package “DescTools” (V0.99.50) [45]. When comparing strand biases of CpG > TpG and non-CpG C > T mutations, a Wilcoxon signed-rank test was used. When comparing the ninety-six individual SBS mutation channels between dMutSα and dMutLα cancers, a Benjamini-Hochberg [46] corrected Wilcoxon *p*-value (P_BHC_) threshold was set at 0.05 to identify significantly altered SBS mutation channels.

## Results

### Patients and their CRCs

We identified 310 MMRd and 1441 MMRp CRCs for inclusion in the study (Methods, Fig. 1). Of these, we identified 20 dMutSα and 164 dMutLα with high-confidence, of which 23 (1.28% of the whole CRC cohort) harboured germline mismatch repair mutations (Lynch Syndrome, Table 1).

**Table 1:** CRC cases in the study. MMRp = mismatch repair-proficient, MMRd = mismatch repair-deficient, Μ = median, IQR = inter-quartile range, ND = Not Disclosed owing to participant confidentiality policies within the 100kGP. *PMS2-*mutant CRCs were excluded from dMutSα/dMutLα classification. NA = Not Applicable

Group	*N*	Lynch syndrome	Female sex	Age (Μ, IQR)	TMB (Μ, IQR)	CpG > TpG Burden (Μ, IQR)	Non-CpG C > T Burden (Μ, IQR)
**MMRp**	1,441	0 (0%)	529 (36.7%)	69 (61–76)	17,483 (14,112–22,841)	2558 (2079–3249)	2782 (2316–3445)
**All MMRd**	310	23 (7.4%)	188 (60.6%)	72 (65–79)	139,682 (110,254–177,942)	18,558 (14,393–25,950)	31,572 (25,217–40,621)
**dMutSα (*MSH2*)**	8	4 (50%)	ND	46 (42–56)	151,404 (117,164–175,894)	27,998 (27,108–33,392)	29,593 (27,520–39,278)
**dMutSα (*MSH6*)**	12	9 (75%)	ND	65 (56–70)	162,972 (118,526–213,208)	39,606 (31,657–55,576)	38,658 (32,151–51,461)
**dMutLα (*MLH1* mutation)**	34	10 (29.4%)	12 (35.3%)	64 (48–71)	118,590 (95,457–149,057)	15,212 (13,169–18,954)	29,443 (24,118–33,444)
**dMutLα (presumed *MLH1* methylation)**	130	0 (0%)	91 (70%)	73 (68–79)	132,605 (106,417–162,697)	17,184 (13,821–23,003)	29,463 (23,949–38,663)
***PMS2-*mutant**	0	0 (0%)	0 (0%)	NA	NA	NA	NA

**Fig. 2:**
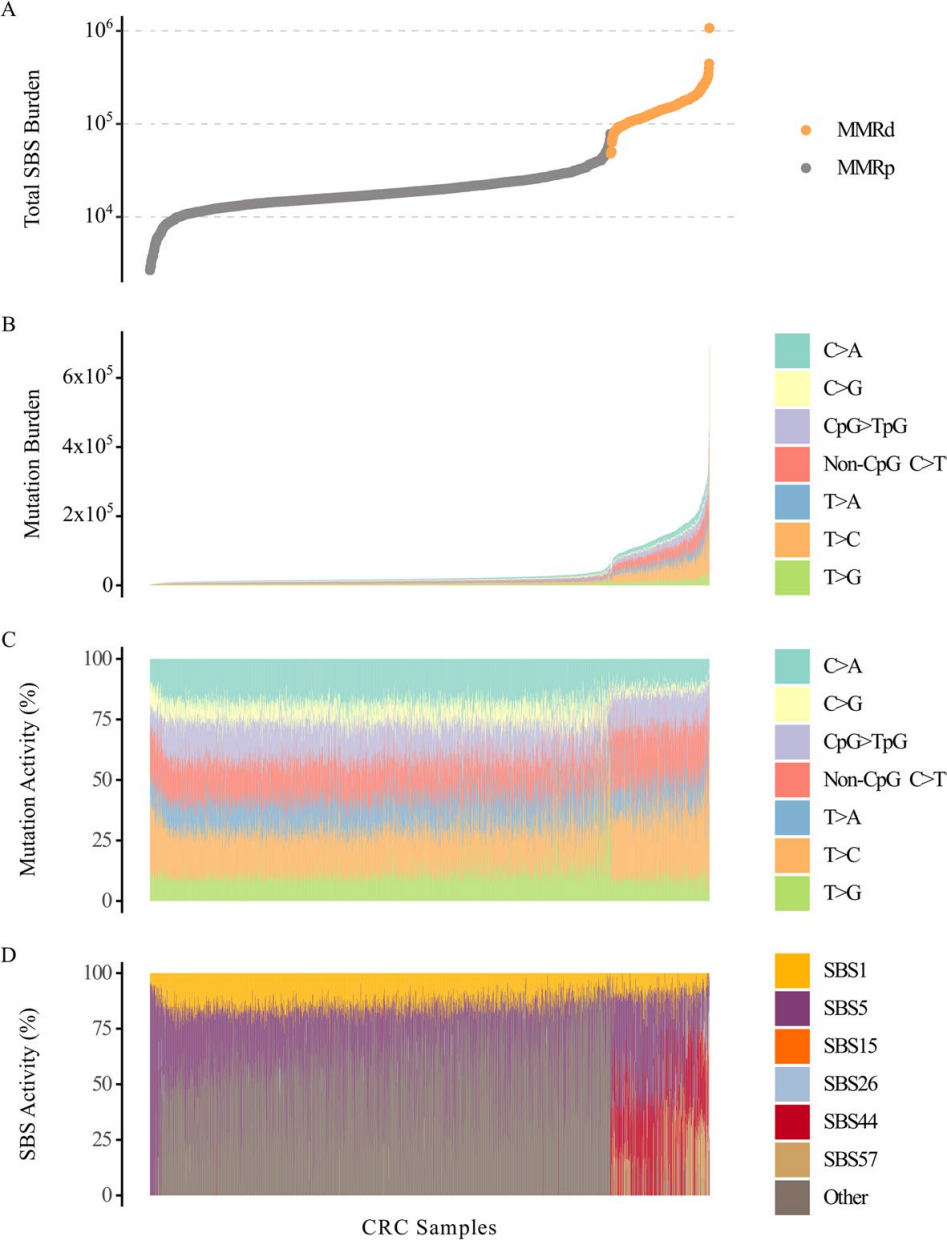
The SBS landscapes of MMRp and MMRd colorectal cancers. The total single base substitution (SBS) burden (**A**) of mismatch repair-proficient (MMRp, grey) and mismatch repair-deficient (MMRd, orange) colorectal cancers. Also shown are the burdens (**B**) and activities (**C**) of the six SBS mutation channels: C > A, C > G, C > T (split into CpG > TpG and non-CpG C > T C > T mutations), T > A, T > C and T > G. **D** The activities of the “clock-like” SBS mutation signatures SBS1 and SBS5, as well as the MMRd-associated signatures SBS15, SBS26, SBS44, and the potential artefact signature SBS57 in the MMRp and MMRd colorectal cancers. Other mutations signatures include SBS2, SBS7c, SBS10a, SBS13, SBS17a, SBS17b, SBS18, SBS28, SBS40, SBS56, SBS93, and SBS94

Single nucleotide (six-channel) SBS mutation burdens (absolute numbers) and proportions in MMRp and MMRd cancers are shown in Fig. 2A–C, Additional file 1: Table S3, and Additional file 2 (Figures S1 & S2). Total SBS burden was about eight-fold higher in MMRd than MMRp tumours (median 139,682 *versus* 17,483; *P* < 2.2 × 10^−16^), with T > C and C > T mutations particularly increased (median 12- and tenfold respectively). Reflecting the burden data, the signatures extracted specifically from MMRd cancers were typified by increased C > T and T > C mutations, including SBS15, SBS26, SBS44, and SBS57 (Fig. 2D; Additional file 1: Table S4). The higher C > T and T > C burdens in MMRd CRCs did not consistently result in higher proportions of these mutations compared with MMRp tumours, owing to the general increase in SBS mutations in the former (Additional file 1: Table S3).

Since the MMRd phenotype of more than 80% of the MMRd cancers was driven by either mutation or putative promoter hypermethylation of *MLH1* (dMutLα), specific features of dMutSα CRCs may have been obscured. We therefore proceeded to analyse dMutSα and dMutLα CRCs separately from the MMRp tumours.

### C > T mutations in dMutSα and dMutLα MMRd CRCs

For both dMutLα and dMutSα, the molecular features of tumour sub-groups were very similar (e.g.* MLH1*-mutation *versus* putative promoter hypermethylation, *MSH2*- *versus MSH6*-mutant, germline *versus* somatic; Additional file 1: Tables S5-S8). Reassuringly, we found that 91% of cancers with putative *MLH1* promoter hypermethylation were located in the proximal colorectum, while 74% also had a co-occurring *BRAF*^*V600E*^ mutation, consistent with previous reports [47, 48].

**Fig. 3:**
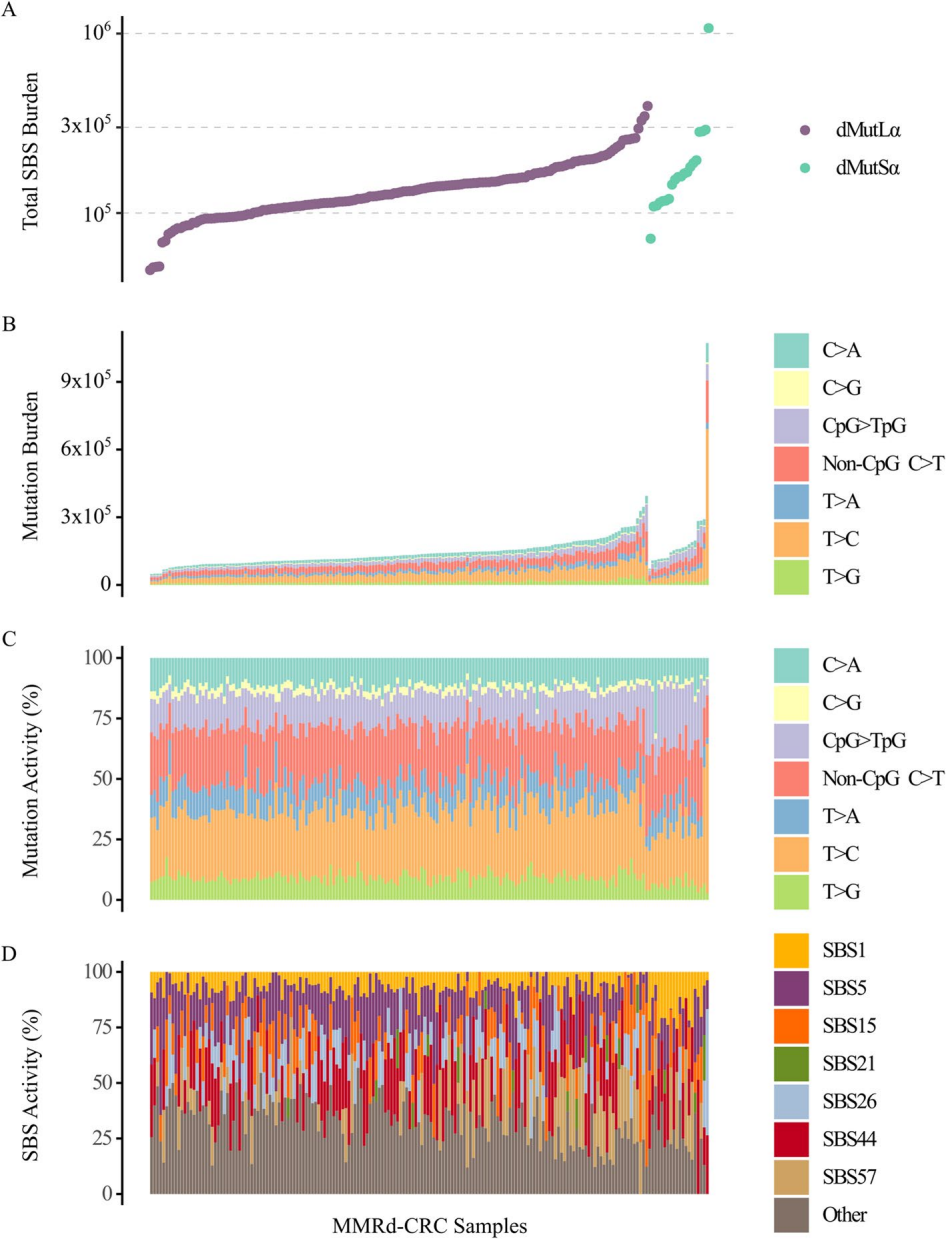
The SBS landscapes of dMutSα and dMutLα colorectal cancers. The total single-base substitution (SBS) burden (**A**) of MutLα-deficient (dMutLα, purple) or MutSα-deficient (dMutSα, green) colorectal cancers. Also shown are the burdens (**B**) and activities (**C**) of the six SBS mutation channels: C > A, C > G, C > T (split into CpG > TpG and non-CpG C > T mutations), T > A, T > C and T > G. **D** The activities of the “clock-like” SBS mutation signatures SBS1 and SBS5, as well as the MMRd-associated signatures SBS15, SBS21, SBS26, SBS44, and the potential artefact signature SBS57 in the dMutLα and dMutSα colorectal cancers. Other mutation signatures include SBS7c, SBS14, SBS17a, SBS18, SBS20, SBS36, SBS41, SBS93, and mutations not assigned to any pre-existing COSMIC mutation signature

**Fig. 4:**
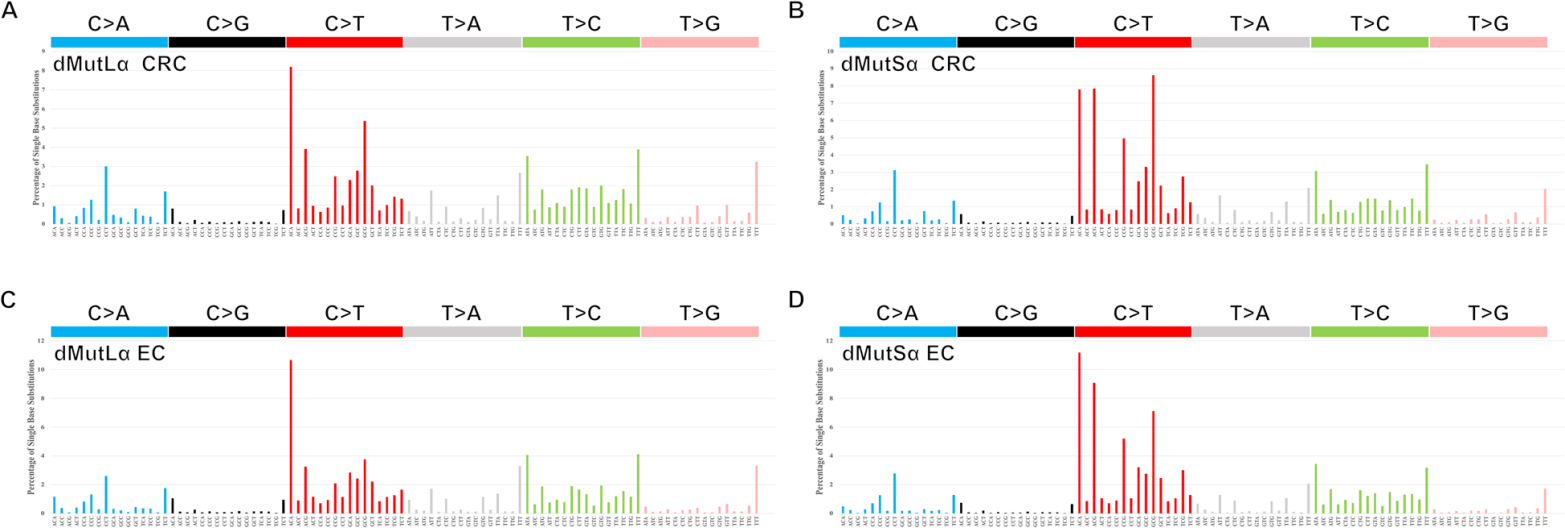
Mutation spectra of dMutLα and dMutSα colorectal and endometrial cancers. The activities (proportions of all 96 SBS channels) of each specific substitution are shown for dMutLα colorectal cancers (**A**), dMutSα colorectal cancers (**B**), and the same for endometrial cancers (**C**,** D**)

We therefore performed a primary comparison (Fig. 3A–D; Table 2; Additional file 2: Figure S3A-H) between the whole dMutSα group (*n* = 20, cancers with *MSH2* or *MSH6* mutations) and the whole dMutLα group (*n* = 164, mostly cancers with presumptive *MLH1* epimutations). The SBS burden of dMutSα CRCs was modestly greater than that of dMutLα tumours (median 159,321 *versus* 130,005, *P* = 0.023). Within the C > T channel, both CpG > TpG and non-CpG C > T burdens were significantly greater in dMutSα CRCs (medians of 33,209 *versus* 16,952; *P* = 1.1 × 10^−8^ for CpG > TpG; and 35,030 *versus* 29,332; *P* = 0.029 for non-CpG C > T, Table 2; Additional file 2: Figure S3D-E). The other five SBS channels showed no significant differences in their mutation burdens. The overall dMutSα and dMutLα mutation spectra were very similar (cosine similarity 0.940), as was the spectrum of the sixteen C > T mutation channels (cosine similarity 0.986; Fig. 4A,B; Additional file 2:Figure S4A-B). The proportion of CpG > TpG mutations was greater in dMutSα CRCs than dMutLα (median 23.9% versus 13.4%; *P* = 1.8 × 10^−7^), whereas the proportions of non-CpG C > T mutations were similar (median 23.4% *versus* 23.2%; *P* = 0.81; Table 2). Aside from C > T mutations, the proportions of the other five SBS channels were greater in dMutLα CRCs than dMutSα tumours, plausibly secondary to the former group’s lower CpG > TpG activity (Table 2).

**Table 2:** Burdens and activities of single-base substitution (SBS) mutations of different types, including SBS signatures, in dMutLα and dMutSα CRCs. Median (IQR) is shown. P from a Wilcoxon test. Other Signatures include SBS7c, SBS14, SBS17a, SBS17b, SBS18, SBS20, SBS36, SBS41, SBS93, and mutations not assigned to any pre-existing COSMIC mutation signature

	**dMutLα (*n* = 164)**	**dMutSα (*n* = 20)**	***P***
**SBS Burden**	130,005 (105,490–157,857)	159,321 (117,164–191,583)	0.023
**Age**	72 (66–79)	57 (48–67)	1.5 × 10^−4^
**C > A Mutation Burden**	14,973 (11,704–18,139)	15,679 (11,613–21,896)	0.54
**C > G Mutation Burden**	3657 (3099–4531)	3415 (2575–4329)	0.084
**CpG > TpG Mutation Burden**	16,952 (13,722–22,623)	33,209 (27,698–45,746)	1.1 × 10^−8^
**Non-CpG C > T Mutation Burden**	29,443 (23,950–38,073)	35,030 (29,016–45,830)	0.029
**T > A Mutation Burden**	13,638 (11,274–17,724)	15,084 (11.639–20,264)	0.43
**T > C Mutation Burden**	36,001 (27,761–48,939)	35,130 (25,750–49,379)	0.94
**T > G Mutation Burden**	11,597 (8637–16,268)	9543 (7470–14,507)	0.22
**C > A Activity (%)**	11.4 (10.1–12.6)	9.5 (8–10.8)	0.0012
**C > G Activity (%)**	2.9 (2.5–3.2)	2.2 (1.7–2.3)	9.8 × 10^−9^
**CpG > TpG Activity (%)**	13.4 (11.5–15.3)	23.9 (18.2–27.9)	1.8 × 10^−7^
**Non-CpG C > T Activity (%)**	23.2 (20.8–25.3)	23.4 (19.6–26.3)	0.81
**T > A Activity (%)**	10.7 (10.2–11.6)	9.1 (8.3–10.3)	4.7 × 10^−5^
**T > C Activity (%)**	28.1 (25.7–31.2)	22.5 (20.9–25.6)	3.9 × 10^−5^
**T > G Activity (%)**	9.2 (7.8–10.2)	6 (5–7.3)	1.4 × 10^−6^
**SBS1 Burden**	8754 (4167–11,671)	25,828 (18,035–33,730)	4.7 × 10^−10^
**SBS1 Activity (%)**	6.4 (3.6–9.2)	18.4 (11.3–21.9)	1.6 × 10^−7^
**SBS5 Activity (%)**	16.4 (10.9–21.6)	14.5 (6.2–18.7)	0.21
**SBS15 Activity (%)**	0 (0–15.4)	5.7 (0–16.5)	0.87
**SBS21 Activity (%)**	0 (0–0)	0 (0–0)	0.35
**SBS26 Activity (%)**	9.3 (0–15.9)	0 (0–0)	0.011
**SBS44 Activity (%)**	21.5 (0–29)	22.5 (12.7–26.7)	0.50
**SBS57 Activity (%)**	0 (0–23.4)	0 (0–17.7)	0.95
**Other Signatures'Activity (%)**	32.7 (24.4–40.6)	23.4 (20–30.6)	0.0085

Mutation signature extraction revealed minimal differences in the prevalence (presence-absence) of C > T- and MMRd-associated mutation signatures in dMutLα and dMutSα CRCs. The only difference was SBS26, composed almost entirely of T > C changes [5], which was more prevalent in dMutLα cancers (present in 53.7% *versus* 15%; *P* = 0.0015; Fisher’s exact test; Additional file 1: Table S9). Analysis of SBS mutation signatures associated with C > T mutations (Table 2; Additional file 2: Figure S4C-F) revealed a higher proportional activity of SBS1 in dMutSα than dMutLα tumours (median 18.4% *versus* 6.4%, *P* = 1.6 × 10^−7^). dMutLα CRCs had a higher proportion of SBS26 (median 9.3% vs 0%; *P* = 0.011) and of a set of miscellaneous, lower activity signatures (median 32.7% *versus* 23.4%; *P* = 0.0085; Fig. 3D; Table 2).

There was no significant difference between the DBS burdens of dMutSα and dMutLα CRCs (median 1871 *versus* 1852; *P* = 0.35; Additional file 1: Table S10; Additional file 2: Figure S5A), although the proportional activity of the MMRd-associated signature DBS7 (main channel TT > AA) was higher in dMutSα tumours (*P* = 6.8 × 10^−9^). Furthermore, the proportion of mutations not assigned to any pre-existing DBS signature was significantly greater in dMutLα CRCs (*P* = 0.02). The total ID burden was not significantly different between dMutSα and dMutLα CRCs (median 228,049 *versus* 302,994; *P* = 0.074; Additional file 1: Table S11; Additional file 2: Figure S5B) and no significant differences in ID signature proportional activity were found (*P* > 0.05).

To confirm that the observed differences between dMutSα and dMutLα CRCs were not a consequence of the mis-classification of cancers with putative *MLH1* promoter hypermethylation as dMutLα, we repeated much of the above analysis for dMutSα *versus MLH1-*mutant CRCs only (Additional file 1: Table S12). Despite the reduced power of this sub-group analysis, we identified most of the same features as in our complete dMutSα *versus* dMutLα analysis (Table 2), including the increased total SBS burden, CpG > TpG burden, and SBS1 activity, with no major discordance.

### Exploring sources of CpG > TpG mutations in dMutSα CRCs

We examined the four trinucleotide mutational channels that together comprise ~ 90% of SBS1 [1, 2, 5], specifically ACG > ATG, GCG > GTG, CCG > CTG and TCG > TTG (Additional file 1: Tables S13 & S14; Additional file2: Figure S4C). We compared the observed mutation numbers in each of the four channels with those in the reference SBS1 signature (41.6%, 21.7%, 24.3%, and 12.4% of CpG > TpG mutations respectively). In both dMutSα and dMutLα CRCs, the burdens of the four CpG > TpG channels across cancers differed significantly from those in SBS1 (*P* < 2.2 × 10^−16^ for both dMutSα and dMutLα; *χ*^2^ test; Additional file 1: Table S14). Specifically, there was a lower proportion of ACG > ATG than in SBS1 (medians 33.4% and 29.6% CpG > TpG mutations in dMutSα and dMutLα respectively), higher GCG > GTG (medians 33.7% and 40.9% respectively), and similar CCG > CTG (21.6% and 18.8% respectively) and TCG > TTG (11.2% and 10.6% respectively).

We searched specifically in dMutSα cancers for a source of the excess GCG > GTG changes, since this might indicate a mechanism other than, or additional to, raised SBS1 activity for the increase in CpG > TpG mutations. This trinucleotide mutation channel is a predominant component of SBS15 (Additional file 2: Figure S4D), which had similarly modest proportional activity in dMutSα and dMutLα CRCs (median 5.7% *versus* 0%, *P* = 0.87), as well as no difference in prevalence in dMutLα and dMutSα CRCs (present in 48.2% *versus* 50%; *P* > 0.99; Fisher’s exact test; Additional file 1: TableS 9). SBS44 (Additional file 2: FigureS4E) also has GCG > GTG as a major component, but again showed no difference in activity between dMutSα and dMutLα CRCs (median 22.5% *versus* 21.5%; *P* = 0.50), while also showing no difference in prevalence (present in 54.9% *versus* 75%; *P* = 0.099; Fisher’s exact test; Additional file 1: Table S9). Thus, while CpG > TpG channels present in other MMRd-associated mutation signatures plausibly contribute the same mutations as in SBS1, we did not identify any lead culprits for the increased CpG > TpG mutations in dMutSα CRCs.

### The dMutSα mutation spectrum can be accounted for by a linear combination of the dMutLα spectrum and SBS1

**Fig. 5:**
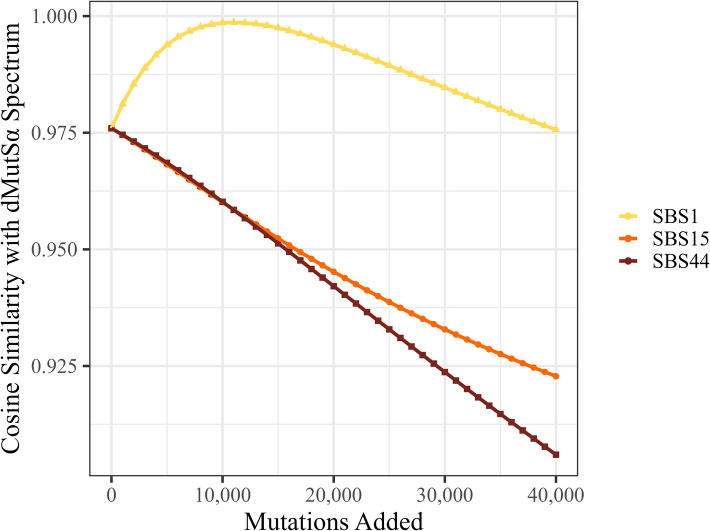
Addition of CpG > TpG mutations in proportion to SBS1 channel activities to the observed dMutLα mutation spectrum causes near-identity to the observed dMutSα spectrum in colorectal cancer. The *y*-axis shows the cosine similarity between the CpG > TpG mutation spectrum of dMutSα CRCs and the same spectrum in dMutLα CRCs when SBS1-associated mutations are proportionally added. As more SBS1 mutations are added (i.e.* x*-axis values increase), the cosine similarity rises to a peak of 0.999. The effects of adding in SBS15 and SBS44 channels are shown for comparison, with no evidence of an effect for either signature

We considered an alternative possibility for the above findings, specifically that the excess GCG > GTG activity over SBS1 reflected a mutational process that has similar activity in both dMutSα and dMutLα cancers. We therefore tested a simple model in which (i) the underlying mutations arising from MMRd, which are most likely replication errors, are the same in dMutSα and dMutLα tumours, and (ii) the differences between dMutSα and dMutLα arise solely from the proposed role of MutSα in the repair of 5’-MeC deamination-associated mutations [14]. We posited that if this were the case, we could re-construct the dMutSα mutation spectrum by adding some unknown level of SBS1-associated mutations on top of the dMutLα SBS mutation spectrum. Since mutational signatures generally contain low-levels of multiple mutation types, we focussed specifically on the four CpG > TpG mutation channels (ACG > ATG, CCG > CTG, GCG > GTG and TCG > TTG). Using the numbers of these mutations in dMutLα CRCs as a baseline, we added extra mutations in these four channels stepwise, in proportion to their relative SBS1 activities (Fig. 5). This had the effect of steadily increasing the cosine similarity with the dMutSα spectrum, based on these four CpG > TpG channels, to a near-perfect level (maximum cosine similarity of 0.999), before falling again. By comparison, adding SBS15- or SBS44-associated mutations led to a monotonic decrease in the similarity to dMutSα. Overall, our modelling suggested that the excess CpG > TpG mutations observed in dMutSα CRCs could plausibly result solely from higher SBS1 activity.

### De novo mutation signature extraction in MMRd CRCs

**Fig. 6:**
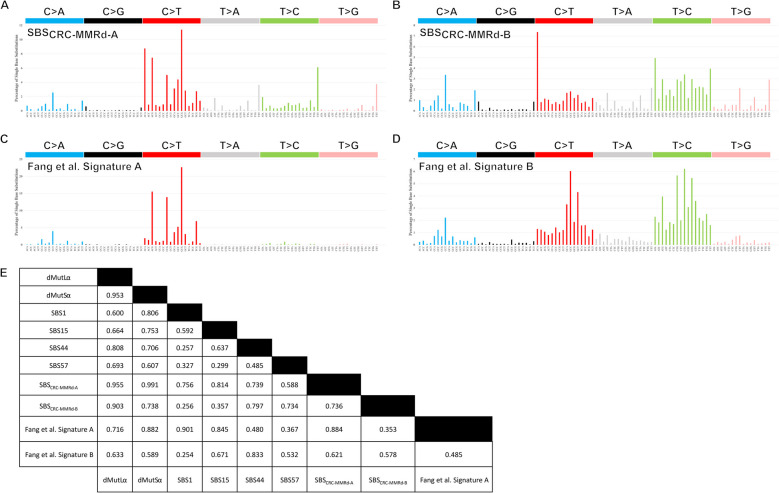
De novo mutation signatures extracted from MMRd colorectal cancers. The activities (proportions of all 96 SBS channels) of each specific substitution are shown for the de novo mutation signatures SBS_CRC-MMRd-A_ (**A**) and SBS_CRC-MMRd-B_ (**B**), extracted from mismatch repair-deficient (MMRd) colorectal cancers. SBS_CRC-MMRd-A_ and SBS_CRC-MMRd-B_, had cosine similarity to one another of 0.675, while the sixteen C > T mutation channels were more similar (cosine similarity 0.736). Also presented are the signature plots approximated from the study by Fang et al. [14], Signature A (**C**) and Signature B (**D**). Also shown are the pairwise cosine similarities of the sixteen C > T mutation channels of these de novo signatures with the dMutLα and dMutSα colorectal cancer spectrum, as well as the spectrum of the COSMIC signatures SBS1, SBS15, SBS44, and SBS57 (**E**). The spectrum of the sixteen C > T mutation channels in SBS_CRC-MMRd-A_ most closely resembled the COSMIC signature SBS15 (cosine similarity 0.814), while SBS_CRC-MMRd-B_ most resembled SBS44 (cosine similarity 0.797)

We wondered whether our previous mutational signature extraction based on unselected CRCs [17] had not fully represented the SBS signatures in MMRd tumours. Thus, we performed de novo SBS mutation signature extraction based solely on our set of dMutSα and dMutLα CRCs (Fig. 6A, B). We limited the total number of de novo signatures to two, termed SBS_CRC-MMRd-A_ and SBS_CRC-MMRd-B_, in order to facilitate comparison with the studies by Fang et al. [14], that was based on multi-cancer exome sequencing data, (Fig. 6C, D; Additional file 1: Table S15; Additional file 2: Figure S4G-H), and by Nemeth et al. [49], which combined whole-genome and whole-exome sequencing data. There was no significant difference in the prevalence of SBS_CRC-MMRd-A_ between dMutLα and dMutSα cancers (present in 100% *versus* 95%; *P* = 0.11; Fisher’s exact test), while SBS_CRC-MMRd-B_ was more prevalent in dMutLα CRCs (present in 98.8% *versus* 80%; *P* = 0.0014; Fisher’s exact test). The proportional activities of C > T mutations in SBS_CRC-MMRd-A_ and SBS_CRC-MMRd-B_ were 53.3% and 22.9% respectively, with corresponding CpG > TpG activities of 26.6% and just 3.4%. T > C mutations comprised 17.9% of SBS_CRC-MMRd-A_ and 39.3% of SBS_CRC-MMRd-B_. The proportional activity of SBS_CRC-MMRd-A_ was significantly higher in dMutSα than dMutLα CRCs (median 75.2% *versus* 44.1%; *P* = 5.7 × 10^−7^), while SBS_CRC-MMRd-B_ was higher in dMutLα CRCs than dMutSα (median 55.9% *versus* 24.8%; *P* = 5.7 × 10^−7^). The sixteen C > T mutation channels in SBS_CRC-MMRd-A_ closely resembled both the dMutSα and dMutLα C > T spectra in our CRCs (cosine similarities 0.991 and 0.955 respectively; Fig. 6E). SBS_CRC-MMRd-B_ was less similar to the dMutSα and dMutLα C > T mutation spectra (cosine similarities 0.738 and 0.903 respectively; Fig. 6E).

There was only modest similarity between our de novo signatures and those of Fang et al. [14] (cosine similarity 0.884 between SBS_CRC-MMRd-A_ and Signature A, and 0.578 between SBS_CRC-MMRd-B_ and Signature B). Specifically, the most active channel in both SBS_CRC-MMRd-A_ and SBS_CRC-MMRd-B_ was ACA > ATA, a channel active in SBS44, in contrast to the very low activity of this channel in the Fang et al. signatures. The reasons for this difference were unclear. They might, for example, be methodological, with Fang et al. [14] using SigFit [50] for mutation signature extraction whereas we used SigProfilerExtractor [30]. We could exclude the higher ACA > ATA levels being a CRC-specific phenomenon, since they were also present in endometrial cancers (see below).

To test whether the differences between our de novo signatures and those of Fang et al. [14] and Nemeth et al. [49] were a consequence of the use of whole-genome *versus* whole-exome sequencing, we extracted two de novo mutation signatures from our MMRd cancers, which we term CRC_Exome-A_ and CRC_Exome-B_, using only mutations within exonic DNA (Additional file 1: Table S15; Additional file 2: Figure S6A-B). The de novo mutation signatures extracted almost completely reproduced those described by Fang et al., with a cosine similarity of 0.984 between CRC_Exome-A_ and Fang et al. Signature A and a cosine similarity of 0.886 between CRC_Exome-B_ and Fang et al. Signature B.

### Assessing potential confounders of associations between MutSα deficiency and C > T substitutions

We investigated a number of features that might confound the observed increase in C > T (including CpG > TpG) mutations in dMutSα CRCs compared to their dMutLα counterparts. First, while SBS1 is a “clock-like” signature [1, 2, 5], dMutSα CRCs were significantly younger than dMutLα cancers (median 57 *versus* 72 years, *P* = 1.5 × 10^−4^; Table 2), indicating that the former's higher CpG > TpG and SBS1 activities were not age-associated. Second, while methylation data from the cancers analysed were not available, dMutLα CRCs are mostly attributed to *MLH1* promoter hypermethylation as part of the CpG island methylator phenotype (CIMP), whereas this feature is not associated with MutSα inactivation [51]. The observed findings thus exist despite the potential CIMP-associated increase in 5’-MeC and hence CpG > TpG changes in dMutLα tumours. Interestingly, however, a significantly smaller proportion of dMutSα CRCs were located within the proximal colorectum compared to dMutLα (70% *versus* 87.8% respectively; *P* = 0.043, Fisher’s exact test). Third, 36.4% of MMRd CRCs harboured at least a mono-allelic truncation in *MBD4* (specifically involved in preventing CpG > TpG mutations), consistent with previous reports [52]. However, of these MMRd CRCs, only 7.1% demonstrated somatic bi-allelic loss of *MBD4* (including second hits by copy number alteration), with no tendency for bi-allelic loss to occur in dMutSα CRCs over dMutLα (*P* > 0.99, Fisher’s exact test). There was no evidence that somatic mutation (either mono-allelic or bi-allelic) in *MBD4* specifically increased CpG > TpG mutation or SBS1 activity in dMutLα (Additional file 1: Table S16) or dMutSα (Additional file 1: Table S17) CRCs. Furthermore, no MMRd CRCs had bi-allelic mutations in the DNA glycosylases *TDG* and *UDG*, involved in the repair of spontaneous deamination of 5’-MeC and unmodified cytosine respectively [53, 54].

It was possible that the increased SBS burden of dMutSα (including CpG > TpG mutation) was a consequence of increased kataegis, localised regions of SBS hypermutation, possibly linked to the APOBEC-induced deamination of single-stranded DNA [20, 55, 56]. Regions of kataegis were identified in 160 (87%) of our MMRd CRCs (18 dMutSα and 142 dMutLα). The number of CpG > TpG mutations mapping to kataegis regions was significantly greater in dMutSα tumours (median 17 *versus* 5, *P* = 0.0023; Additional file 1: Table S18), which was complemented by increased activity of CpG > TpG mutations in these regions compared to dMutLα tumours (median 11% *versus* 6.8%, *P* = 8.3 × 10^−4^; Additional file 1: Table S18). In line with genome-wide data, the burden of non-CpG C > T mutations in kataegis regions was significantly greater in dMutSα tumours (median 29 *versus* 16, *P* = 0.029, while the activity of these mutations was not (median 23.6% *versus* 21.6%, *P* = 0.35; Additional file 1:Table S18). However, when compared to the whole genome, the activity of CpG > TpG mutations was significantly lower in kataegis regions in both dMutSα (median 11% *versus* 23.6%, *P* = 3.3 × 10^−4^; Wilcoxon signed-rank test) and dMutLα (median 6.8% *versus* 13.2%, *P* < 2.2 × 10^−16^; Wilcoxon signed-rank test). Thus, increased mutation in kataegis regions could not explain the increased CpG > TpG mutations in dMutSα CRCs.

### Relationship of C > T mutations to DNA methylation, replication timing, and transcriptional strand

In order to obtain more information regarding the origins of C > T mutations in dMutSα and dMutLα CRCs, we annotated their locations with methylation state, replication timing, transcription strand, and replication strand. The 100kGP sequencing data permitted the assessment of these mutations throughout the genome. In brief, the findings were as follows:

(a) CpG > TpG mutations were more common at highly-methylated CpG sites in all CRC types, including MMRp, MMRd, *POLE*-mutant, and *POLD1-*mutant (*P* = 0.001 in all cases; JT test; Additional file 2: Figure S7A). This is consistent with previous reports [57, 58] and was apparent in dMutSα (*P* = 0.001; JT test) and dMutLα (*P* = 0.003, JT test) CRCs when analysed separately. However, the strength of this association (regression slope) was significantly greater in dMutSα CRCs than dMutLα (1245 *versus* 340; *P* = 1.81 × 10^−4^; Additional File 2: Figure S7B). This may simply reflect the strong co-variation between dMutLα and CIMP, and hence higher background methylation levels in the dMutLα tumours that dampen any association signal.

(b) In all CRC types, CpG > TpG mutations were associated with late-replicating DNA, with similar effects in dMutSα and dMutLα tumours, although the former had a higher background level of mutation (regression intercept 1023 *versus* 475; Additional file 2: Figures S7C-D).

(c) Evidence of mild transcriptional strand bias of non-CpG C > T mutations was present, but this was similar in dMutSα and dMutLα tumours (median log_2_(Coding/Template) ratio − 0.127 *versus* − 0.124; *P* = 0.86; Additional file 1: Tables S19 & S20; Additional file 2: Figure S8A).

### Relationship of C > T mutations to the DNA replication strand

**Fig. 7:**
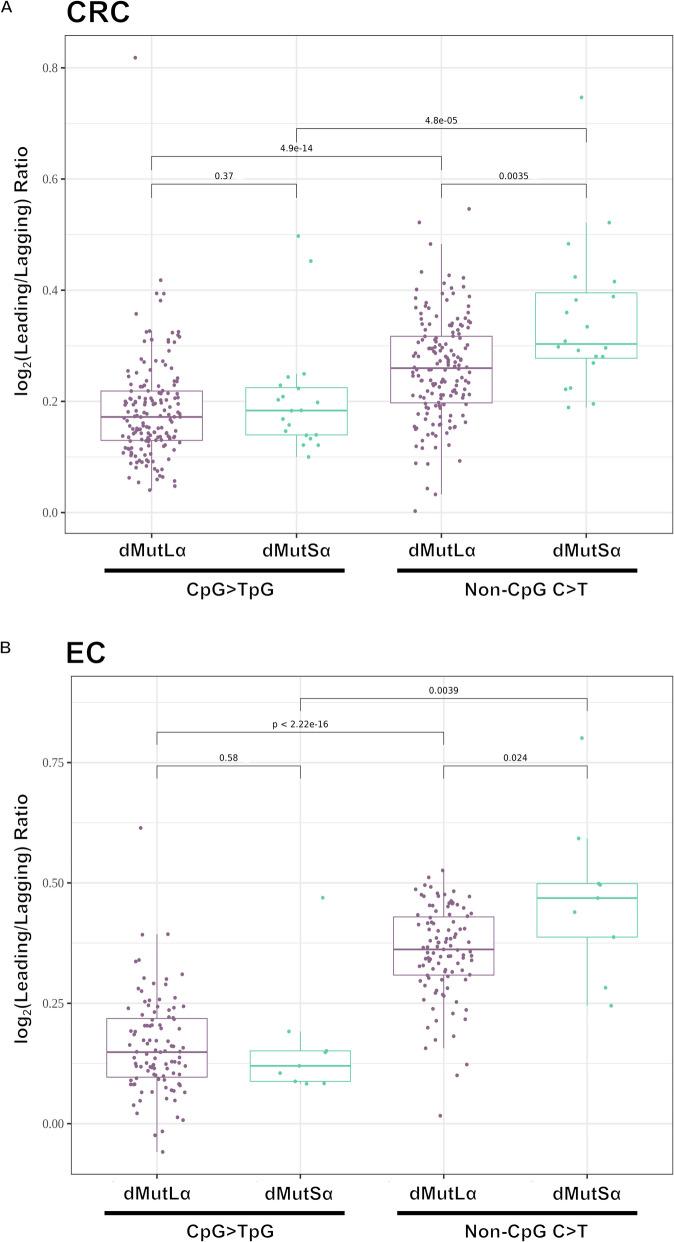
Replication strand bias of C > T mutations in MMRd CRC and EC. The replication strand (log_2_(Leading/Lagging)) bias of CpG > TpG and non-CpG C > T mutations in mismatch repair-deficient (MMRd) colorectal cancers (**A**) and endometrial cancers (**B**), classified as MutLα-deficient (dMutLα, purple) or MutSα-deficient (dMutSα, green). P comparing dMutLα to dMutSα from a Wilcoxon test. P comparing CpG > TpG *versus* non-CpG C > T from a Wilcoxon signed-rank test

We initially confirmed that, in *POLE*-mutant CRCs, there was a significant excess of both CpG > TpG (median 27,092 *versus* 14,126; *P* = 7.6 × 10^−6^; Wilcoxon signed-rank test) and non-CpG C > T (median 29,282 *versus* 16,045; *P* = 7.6 × 10^−6^; Wilcoxon signed-rank test) mutations on the leading strand, consistent with these mutations arising from unrepaired DNA replication errors, as expected. We also confirmed that no replication strand bias existed in a set of *MBD4*-mutant colorectal polyps (Additional file 1: Table S21), consistent with the very large number of CpG > TpG changes in those tumours being independent of DNA replication [8]. In the MMRd CRCs, there were significantly more CpG > TpG mutations on the leading than lagging strand (Additional file 1: Table S21). This association was present in both dMutSα (median 6704 *versus* 5959; *P* = 1.9 × 10^−6^; Wilcoxon signed-rank test; Table 3) and dMutLα (median 3428 *versus* 2987; *P* < 2.2 × 10^−16^; Wilcoxon signed-rank test; Table 3) tumours. There were very similar log_2_(Leading/Lagging) ratios in the two MMRd sub-types (median 0.184 *versus* 0.172; *P* = 0.37; Fig. 7A), contrary to previous reports from multi-cancer exome data [14]. Non-CpG C > T mutations were also more prevalent on the leading strand in both dMutSα (median 7331 *versus* 5729; *P* = 1.9 × 10^−6^; Wilcoxon signed-rank test; Table 3) and dMutLα (median 5,827 *versus* 4909; *P* < 2.2 × 10^−16^; Wilcoxon signed-rank test; Table 3) CRCs. This leading strand bias was in keeping with previous reports [14], which found a leading strand bias for four of the six SBS channels in MMRd cancer, the exceptions being C > A and T > A. The leading strand bias of non-CpG C > T mutations was significantly greater than that of CpG > TpG mutations in both dMutSα (median 0.303 *versus* 0.184; *P* = 4.8 × 10^−5^; Wilcoxon signed-rank test) and dMutLα (median 0.260 *versus* 0.172; *P* = 4.9 × 10^−14^; Wilcoxon signed-rank test) cancers, consistent with previous reports [14].

**Table 3:** CpG > TpG and non-CpG C > T mutations on the leading or lagging replication strand in dMutLα and dMutSα colorectal cancers. Median (IQR) shown. P_Leading*versus*Lagging_ from Wilcoxon signed-rank test. P_dMutSα *versus*dMutLα_ from Wilcoxon test

		**Leading strand mutations**	**Lagging strand mutations**	**log**_**2**_**(Leading/Lagging)**	**P**_**Leading*versus*Lagging**_	**P**_**dMutSα *versus*dMutLα**_
**CpG > TpG**	**dMutLα (*n* = 164)**	3428 (2761–4489)	2987 (2440–4034)	0.172 (0.130–0.219)	< 2.2 × 10^−16^	0.37
	**dMutSα (*n* = 20)**	6704 (5412–9067)	5959 (4935–7853)	0.184 (0.140–0.225)	1.9 × 10^−6^	
**Non-CpG C > T**	**dMutLα (*n* = 164)**	5827 (4785–7822)	4909 (4078–6249)	0.260 (0.197–0.317)	< 2.2 × 10^−16^	0.0035
	**dMutSα (*n* = 20)**	7331 (5908–9617)	5729 (4835–7891)	0.303 (0.278–0.395)	1.9 × 10^−6^	
**All SBS mutations**	**dMutLα (*n* = 164)**	22,723 (18,386–27,739)	24,992 (20,237–31,192)	− 0.135 (− 0.177 to − 0.103)	< 2.2 × 10^−16^	2.8 × 10^−5^
	**dMutSα (*n* = 20)**	28,424 (21,530–34,515)	30,638 (22,400–36,580)	− 0.048 (− 0.094 to − 0.015)	8.5 × 10^−4^

If the excess CpG > TpG mutations observed in dMutSα CRCs were entirely due to unrepaired deamination of 5’-MeC (SBS1-associated), we would have expected a log_2_(Leading/Lagging) ratio closer to zero than that observed in dMutLα CRCs. As seen in Fig. 5, almost perfect cosine similarity of the CpG > TpG spectrum of dMutSα and dMutLα CRCs was achieved when dMutSα comprised a mixture of 61% dMutLα and 39% SBS1. If the latter were the result of replication-independent deamination of 5’-MeC, while the rest were replication, we estimated the expected log_2_(Leading/Lagging) ratio of CpG > TpG mutations to be 0.112 in dMutSα CRCs. However, the observed strand bias of dMutSα was significantly greater than this (*P* = 9.5 × 10^−6^, Wilcoxon signed-rank test), strongly suggesting that at least some of the excess CpG > TpG mutations in dMutSα CRCs are replication-derived.

### dMutSα endometrial cancers show increased C > T mutations and SBS signatures with similar features to those in CRCs

Since endometrial cancers (ECs) are frequently dMutSα or dMutLα, we analysed C > T mutations in a cohort of 596 primary, treatment-naïve ECs from the 100kGP that met the same inclusion criteria used for CRCs. In total, 360 (60.4%) ECs were MMRp and 157 (26.3%) MMRd (Methods; Table 4; Additional file 2: Figure S9). After excluding 45 tumours with an uncertain cause of MMRd, 9 were classified as dMutSα and 103 as dMutLα, similar proportions to those in CRC (Table 4). MMRd ECs had a seven-fold increase in SBS burden compared to MMRp cancers (median 61,840 *versus* 9115; *P* < 2.2 × 10^−16^), and at the individual SBS channel level, the T > C mutation burden showed an eight-fold median increase in MMRd ECs (Additional file 1: Table S22; Additional file 2: Figures S10 & S11). CpG > TpG mutations were increased six-fold, while non-CpG C > T mutations showed a nine-fold increase. In contrast to CRCs, the most prevalent MMRd-associated signature in ECs was SBS15 (present in 41.4% of MMRd ECs), while SBS44 was absent (Additional file 1: Table S23; Additional file 2: FigureS10D).

**Table 4:** EC cases in the study. MMRp = mismatch repair-proficient, MMRd = mismatch repair-deficient, M = median, IQR = inter-quartile range**,** ND = Not Disclosed owing to participant confidentiality policies within the 100kGP. *PMS2-*mutant ECs were excluded from dMutSα/dMutLα classification. NA = Not Applicable

Group	*N*	Lynch syndrome	Age (Μ, IQR)	TMB (Μ, IQR)	CpG > TpG Burden (Μ, IQR)	Non-CpG C > T Burden (Μ, IQR)
**MMRp**	360	0 (0%)	68 (60–74)	9115 (7304–11,416)	1188 (925–1483)	1872 (1531–2329)
**All MMRd**	157	5 (3.18%)	68 (61–76)	61,840 (47,910–85,227)	6774 (4803–10,197)	17,171 (13,114–21,787)
**dMutSα (*MSH2*)**	5	1 (20%)	55 (52–57)	105,702 (98,503–113,452)	24,240 (18,434–25,304)	27,221 (22,965–29,992)
**dMutSα (*MSH6*)**	4	3 (75%)	ND	81,556 (74,725–88,830)	21,880 (21,375–22,179)	23,474 (20,349–25,633)
**dMutLα (*MLH1* mutation)**	3	1 (33.3%)	ND	95,035 (79,204–96,890)	10,146 (10,025–11,585)	22,614 (20,047–23,113)
**dMutLα (presumed *MLH1* methylation)**	100	0 (0%)	69 (62–76)	58,331 (46,995–79,656)	6108 (4677–8323)	16,651 (13,398–20,873)
***PMS2-*mutant**	0	0 (0%)	NA	NA	NA	NA

The EC analysis supported the CRC data throughout, with only very minor differences (details in Figs. 4C, D and 7B, Additional file 1: TablesS22-S39, Additional file 2: FiguresS4, S8B & S10-S18, and Additional file 3). Briefly, dMutSα ECs displayed an increased SBS burden compared with dMutLα ECs (median 96,264 *versus* 59,497; *P* = 6.9 × 10^−4^), mostly driven by an increase in CpG > TpG mutation (median 22,079 *versus* 6,274; *P* = 1.2 × 10^−6^); this was accompanied by an increase in SBS1 activity (median 20.9% *versus* 5.4%; *P* = 1.6 × 10^−4^; Additional file 1: Table S28). Just as in CRC, the dMutSα C > T spectrum in ECs could be reconstituted when SBS1 mutations were added to the dMutLα spectrum (Additional file 2: Figure S17). There was also a significant leading strand bias of CpG > TpG mutations in both dMutLα (median 1196 *versus* 1095; *P* < 2.2 × 10^−16^) and dMutSα (median 4366 *versus* 4009; *P* = 0.0039) ECs (Additional file 1: Table S39), while the log_2_(Leading/Lagging) ratio of mutations was not different between dMutSα and dMutLα cancers (median 0.120 *versus* 0.149; *P* = 0.58; Fig. 7B), as also seen in CRCs.

### Could MutSα play a role in detecting base mismatches other than T:G outside the context of DNA replication?

MutSα detects multiple types of base mismatch during DNA replication [59]. If it also detects T:G mismatches caused by deamination of 5’-MeC outside DNA replication, as proposed [14], that role could extend to other T:G mispairs (for example, methyl-G:T), or indeed to other types of mismatch that arise outside DNA replication. To this end, we compared the activities of exemplar mutation signatures associated with replication-independent DNA damage processes in dMutSα and dMutLα cancers [59–61].

Mutation signature SBS18 is attributed to reactive oxygen species-induced DNA damage [6, 62], principally the creation of 8-oxo-guanine. Resulting DNA mismatches, such as 8-oxo-G:A, are resolved by the DNA base excision repair pathway [61]. C > A mutations comprise ~ 54% of SBS18-associated changes, with particularly notable channels including TCT > TAT (activity 12.3%), GCA > GAA (10.9%), TCA > TAA (7.4%) and CCA > CAA (7.3%) [5]. SBS18 was absent in dMutLα and dMutSα ECs, while there was no difference in the prevalence of this signature in dMutLα and dMutSα CRCs (present in 0.54% *versus* 0%; *P* > 0.99; Fisher’s exact test). Overall, C > A burdens were similar in dMutSα and dMutLα CRCs (median 15,679 *versus* 14,973; *P* = 0.54; Fig. 3B; Table 2), but a little increased in dMutSα over dMutLα ECs (median 8057 *versus* 6161; *P* = 0.03; Additional file 1: Table S28; Additional file 2: Figure S13B). C > A mutation activity was higher in dMutLα CRCs (median 11.4% *versus* 9.5%; *P* = 0.0012; Fig. 3C; Table 2) and ECs (median 11% *versus* 8.7%; *P* = 3.6 × 10^−5^; Additional file 1: Table S28; Additional file 2: Figure S13C), plausibly reflecting higher C > T in the latter. The burdens of the four major SBS18-associated mutations described above were either not significantly different between dMutSα and dMutLα, or significantly greater in dMutLα cancers in both MMRd CRCs (Additional file 1: Table S14) and ECs (Additional file 1: Table S32), suggesting that there is no critical role for the MutSα complex in the prevention of these mutations. Furthermore, the activities of four SBS18-associated channels were significantly greater in dMutLα CRCs and ECs (Additional file 1: Tables S14 & S32).

Other replication-independent sources of base mismatches include TCN > TTN (associated with SBS2) and TCN > TGN (associated with SBS13), resulting from APOBEC-induced cytidine deamination [63, 64]. In both MMRd CRCs and ECs, signatures SBS2 and SBS13 were absent in dMutLα and dMutSα cancers, consistent with reports of a requirement for intact MMR and APOBEC3-induced mutation [65]. With the exception of TCG > TTG, the burdens of all SBS2- and SBS13-associated mutation channels were not significantly different between dMutSα and dMutLα CRCs (Additional file 1: Table S14). However, while the burdens of three of the four TCN > TTN mutations were increased in dMutSα ECs, the exception being TCT > TTT, the proportional activities of these channels were either not different between dMutSα and dMutLα cancers or greater in dMutLα cancers, with the exception of TCG > TTG (Additional file 1: Table S32). This suggests that MutSα deficiency does not drive an increase in SBS2- or SBS13-associated mutation compared to other MMRd cancers.

Overall, the data do not suggest a general role for the MutSα complex in the correction of DNA mismatches outside the context of DNA replication. However, a special role for MutSα in detecting T:G mismatches from CpG > TpG mutations remains possible, given that this pairing is unusual in not involving modified or atypical bases.

## Discussion

We found that C > T and T > C changes are the most increased SBS mutations in MMRd CRCs and ECs compared with their MMRp counterparts. This was reflected in the increased activities of SBS mutational signatures with prominent C > T and T > C components in both dMutSα and dMutLα CRCs and ECs, including signatures SBS15, SBS26, SBS44, and SBS57. dMutSα CRCs had a higher SBS burden than dMutLα tumours, and higher proportional C > T activity, mostly owing to CpG > TpG changes. Whereas most hyperactive mutational processes were shared among MMRd cancers, the activity of signature SBS1, conventionally linked to spontaneous deamination of 5’-MeC, rather than DNA replication repair, was specifically increased in dMutSα tumours. The differences between the burden and spectra of C > T mutations in dMutSα and dMutLα cancers could be explained fully and precisely by an admixture model, in which the dMutSα CpG > TpG spectrum was composed of 61% dMutLα-associated mutations and 39% additional SBS1-type mutations.

Despite these findings, CpG > TpG mutations associated with MMRd showed bias to the leading strand in DNA replication, suggesting at least a partial origin in DNA replication rather than deamination of 5’-MeC. Previous studies suggested that the leading strand bias of CpG > TpG mutations was less in dMutSα cancers than dMutLα [14]. Here, leading strand bias was detected for both CpG > TpG and non-CpG C > T mutations in both dMutSα and dMutLα CRCs and ECs. Importantly, and contrary to previous reports, dMutSα and dMutLα CRCs and ECs had similar log_2_(Leading/Lagging) ratios for CpG > TpG mutations. Since the median number of CpG > TpG mutations in dMutSα tumours was almost double that in dMutLα CRCs and almost four-fold higher in dMutSα ECs, had that CpG > TpG excess come from deamination of 5’-MeC, it should have been detectable as a decrease in the replication strand ratio. Indeed, the strand bias was significantly greater than that predicted by the admixture model. As a control, we found that SBS1 mutations in what is arguably their purest form, namely unrepaired deamination of 5’-MeC in patients with germline MBD4 deficiency [8], were associated with a mutation signature closely resembling SBS1 and almost entirely comprised of CpG > TpG mutations [7, 9], but showed no association with DNA replication strand. While Fang et al. [14] did observe a reduced replication strand bias for CpG > TpG mutations in dMutSα cancers compared to dMutLα, they did not rule out a replication-dependent mechanism for at least some CpG > TpG mutations in dMutSα tumours.

Our study supports many of the findings of Fang et al. [14], who first alighted on the importance of C > T changes in dMutSα cancers. Our principal limitation, compared with TCGA-COADREAD [29] data used by Fang et al. [14], is the lack of RNA-sequencing and DNA methylation data within the 100kGP, which mandated that we assign the cause of MMRd in each CRC and EC using DNA-sequencing data alone. *MLH1* methylation was thus allotted by stringent exclusion of other causes of MMRd. Nevertheless, the assignment of cancers as dMutSα or dMutLα had comparable performance to other studies [14] and was supported by analyses based on the sub-group of CRCs that had dMutSα or dMutLα status originating from bi-allelic mutations (including substitutions, indels, and copy number changes). 100kGP also has a number of advantages over previous work in this field, including a comprehensive set of criteria for patient recruitment with very few exclusions, reducing any bias toward a younger age profile than the cancer population as a whole; large sample sets of the two major MMRd cancer types, reducing heterogeneity compared with pan-cancer data; use of WGS data, with benefits for statistical power, accurate mutation calling, unbiased mutation spectra, signature assignment, and links to other genomic features; and consistent sample preparation, molecular methods, and analytical pipelines within and between cancer types.

dMutSα was not associated with other mutational processes or signatures associated with base mismatches (e.g. oxidative damage or APOBEC). The question thus arises as to why only T:G mismatches are found by dMutSα, at least outside replication. One possibility is that most other mismatches involve modified bases, such as 8-oxoguanine. However, dMutSα tumours also had a higher burden of non-CpG C > T changes than dMutLα tumours. Is it possible that MutSα is also involved in the detection of U:G mismatches? Or could the excess of both CpG > TpG and non-CpG C > T changes in dMutSα tumours have their origins in replication-induced or -associated changes, such as 5’-MeC:A mismatches, that mimic SBS1 in their effects [41, 42]? The latter alternative explanation is consistent with published data demonstrating that both the expression and activity of the MutSα and MutLα complexes are highest in S- and G_2_-phase of the cell cycle [66], while there is no such cell cycle-specific regulation of proteins involved in the repair of 5’-MeC deamination events, such as MBD4, that are ubiquitously expressed throughout the cell cycle [67, 68]. C:A mismatches in DNA replication could, for example, give rise to both the C > T and T > C excesses seen in MMRd tumours, and these mispairs might be favoured by 5’-MeC more than unmodified cytosine. Although circumstantial evidence, it is also notable that deficiency of proofreading deficiency by leading strand DNA polymerase *POLE* (but not *POLD1*) causes a large excess of CpG > TpG mutations, manifested mostly as TCG > TTG changes within signature SBS10b. Furthermore, it has previously been reported [41, 42] that *POLE* is up to seven times more error-prone when replicating a template 5’-MeC, possibly resulting in C:A DNA mismatches, which can be propagated into CpG > TpG mutations. However, in vitro data on replication errors opposite 5’-MeC in MMRd cancers are lacking. The question of why MutSα would be more affected by CpG > TpG changes than MutLα also remains open, but we speculate that there is some redundancy in the latter (for example, BER could correct some replication-associated errors, since they will persist unless corrected in the next S-phase, while no compensatory redundancy in error detection exists). This was evidenced by the study by Goellner et al. [69], who demonstrated an interaction between Msh2 and Exo1 in *S. cerevisiae*, suggesting that mismatch repair can be initiated in a MutLα-independent manner.

## Conclusions

In conclusion, dMutSα CRCs and ECs are more prone than their dMutLα counterparts to acquire C > T mutations, the excess being mostly CpG > TpG changes that resemble those in COSMIC signature SBS1. Formally, we have shown that adding SBS1-associated mutations to the dMutLα mutational spectrum in an admixture model can result in near-identity to the dMutSα spectrum. Fang et al. [14] suggested that the cause of the extra C > T changes in dMutSα cancers is a non-canonical role for the MutSα complex in detecting T:G mismatches caused by deamination of 5’-MeC throughout the cell cycle. Our data broadly support the observations of Fang et al. [14], and we agree that their proposed explanation is both original and plausible. However, some of our additional findings, especially the similar leading replication strand bias of CpG > TpG mutations in dMutSα and dMutLα tumours and the excess of non-CpG C > T mutations in dMutSα tumours, suggest that other explanations remain possible. Specifically, the elevated number of CpG > TpG mutations in dMutSα tumours might originate from undetected (and hence unrepaired) DNA mismatches caused by replication errors. Tomkova et al. [41, 42] have recently proposed that the replication fidelity of the replicative DNA polymerase ε is reduced when replicating a template 5’-MeC, potentially driving an increase in CpG > TpG mutation should these replication errors go unrepaired in dMutSα tumours. Inherent differences in the roles of MutSα (detection) and MutLα (repair) in replication-coupled DNA mismatch repair may explain differences between these MMRd groups and warrant further investigation.

## Supplementary Information

Additional file 1: Ward_et_al_Additional_File_1.xlsx. Tables S1-S39, including their associated legends

Additional file 2: Ward_et_al_Additional_File_2.docx. Figures S1-S18, including their associated legends

Additional file 3: Ward_et_al_Additional_File_3.docx. Analysis of 100kGP endometrial cancers

## Data Availability

Research on the de-identified patient data used in this publication can be carried out in the Genomics England Research Environment subject to a collaborative agreement that adheres to patient led governance. All interested readers will be able to access the data in the same manner that the authors accessed the data. For more information about accessing the data, interested readers may contact research-network@genomicsengland.co.uk or access the relevant information on the Genomics England website: https://www.genomicsengland.co.uk/research. The application process for accessing 100kGP data is estimated to take two to three weeks, subject to the applicant being verified by their institution and the completion of all necessary training.Research on the de-identified patient data used in this publication can be carried out in the Genomics England Research Environment subject to a collaborative agreement that adheres to patient led governance. All interested readers will be able to access the data in the same manner that the authors accessed the data. For more information about accessing the data, interested readers may contact research-network@genomicsengland.co.uk or access the relevant information on the Genomics England website: https://www.genomicsengland.co.uk/research. The application process for accessing 100kGP data is estimated to take two to three weeks, subject to the applicant being verified by their institution and the completion of all necessary training.

## Abbreviations

5’-MeC: 5’-Methylcytosine
100kGP: UK 100,000 Genomes Project
BER: Base excision repair
COSMIC: Catalogue of Somatic Mutations in Cancer
CpG > TpG: C > T mutations at CpG sites
CRC: Colorectal cancer
DBS: Doublet-base substitution
dMutLα: MutLα-deficient
dMutSα: MutSα-deficient
EC: Endometrial cancer
EDM: Exonuclease domain mutation
ID: Insertion-deletion
JT test: Jonckheere-Terpstra non-parametric test for trend
MBD4: Methyl-CpG binding domain 4
MLH1: MutL homologue 1
MMRd: Mismatch repair-deficient
MMRp: Mismatch repair-proficient
MSH2: MutS homologue 2
MSH6: MutS homologue 6
MSI: Microsatellite instability
mSINGS: Detecting Microsatellite Instability by Next-Generation Sequencing
Non-CpG C > T: C > T mutations outside CpG sites
POLD1: DNA polymerase δ
POLE: DNA polymerase ε
SBS: Single-base substitution
TCGA-COADREAD: The Cancer Genome Atlas (Colorectal & Rectal Adenocarcinoma domains)
TDG: Thymine DNA glycosylase
UDG: Uracil DNA glycosylase
WGS: Whole-genome sequencing
