# Supplementary material for: Replication-associated mechanisms contribute to an increased CpG > TpG mutation burden in mismatch repair-deficient cancers
Source: Genome Med. 2025 Aug 25;17:95. doi: 10.1186/s13073-025-01525-6 (PMC12376743; doi:10.1186/s13073-025-01525-6)
Supplement: Supplementary file 2 — Additional file 2: Ward_et_al_Additional_File_2.docx. Figures S1-S18, including their associated legends [file 13073_2025_1525_MOESM2_ESM.docx]

**Figure S1. The SBS mutation burden of MMRp and MMRd colorectal cancers.** The total single-base substitution (SBS burden of mismatch repair-proficient (MMRp, grey) and mismatch repair-deficient (MMRd, orange) colorectal cancers (A). Also shown are the burdens of the specific mutation channels C>A (B), C>G (C), CpG>TpG (D), Non-CpG C>T (E), T>A (F), T>C (G) and T>G (H).


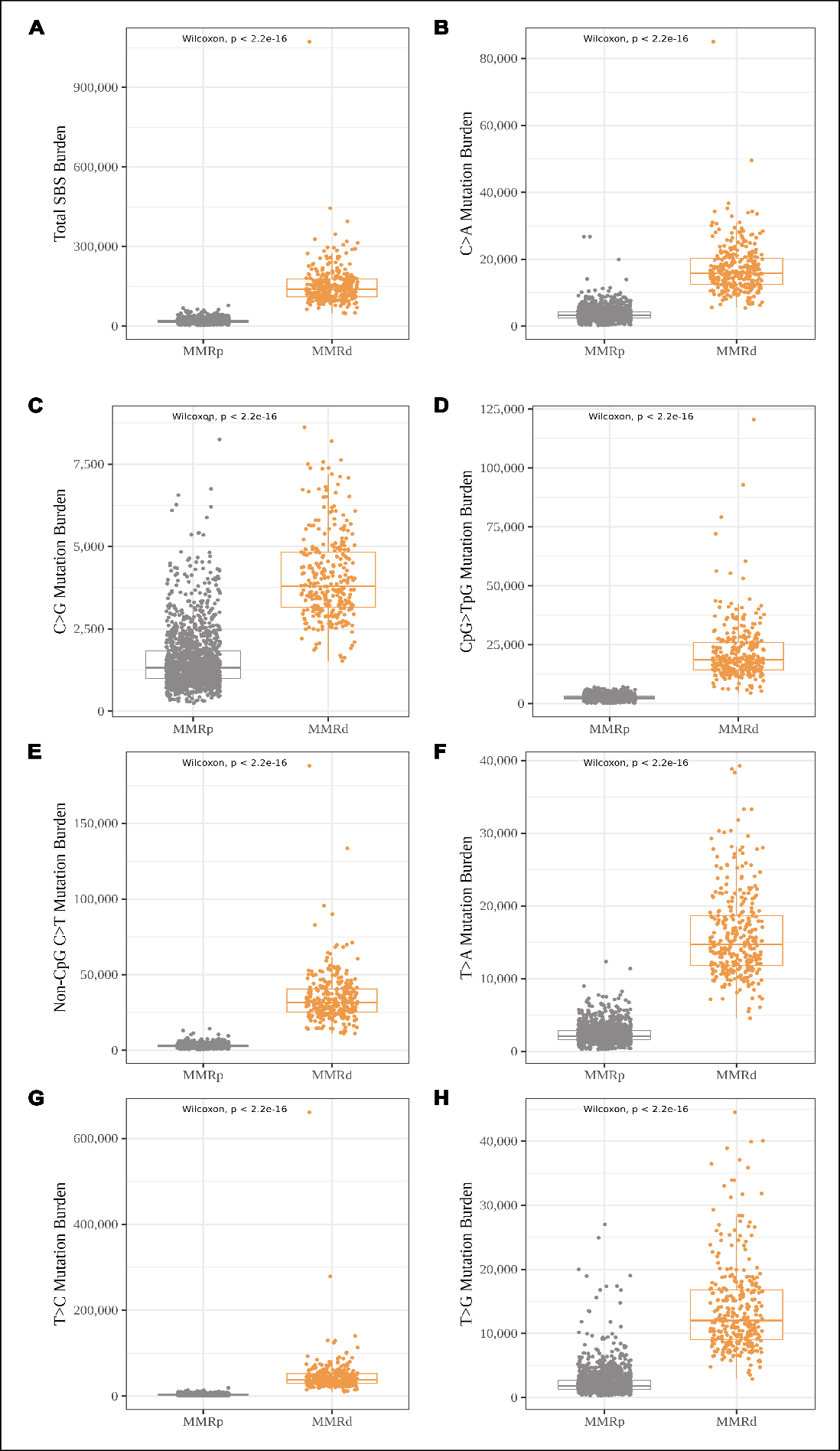


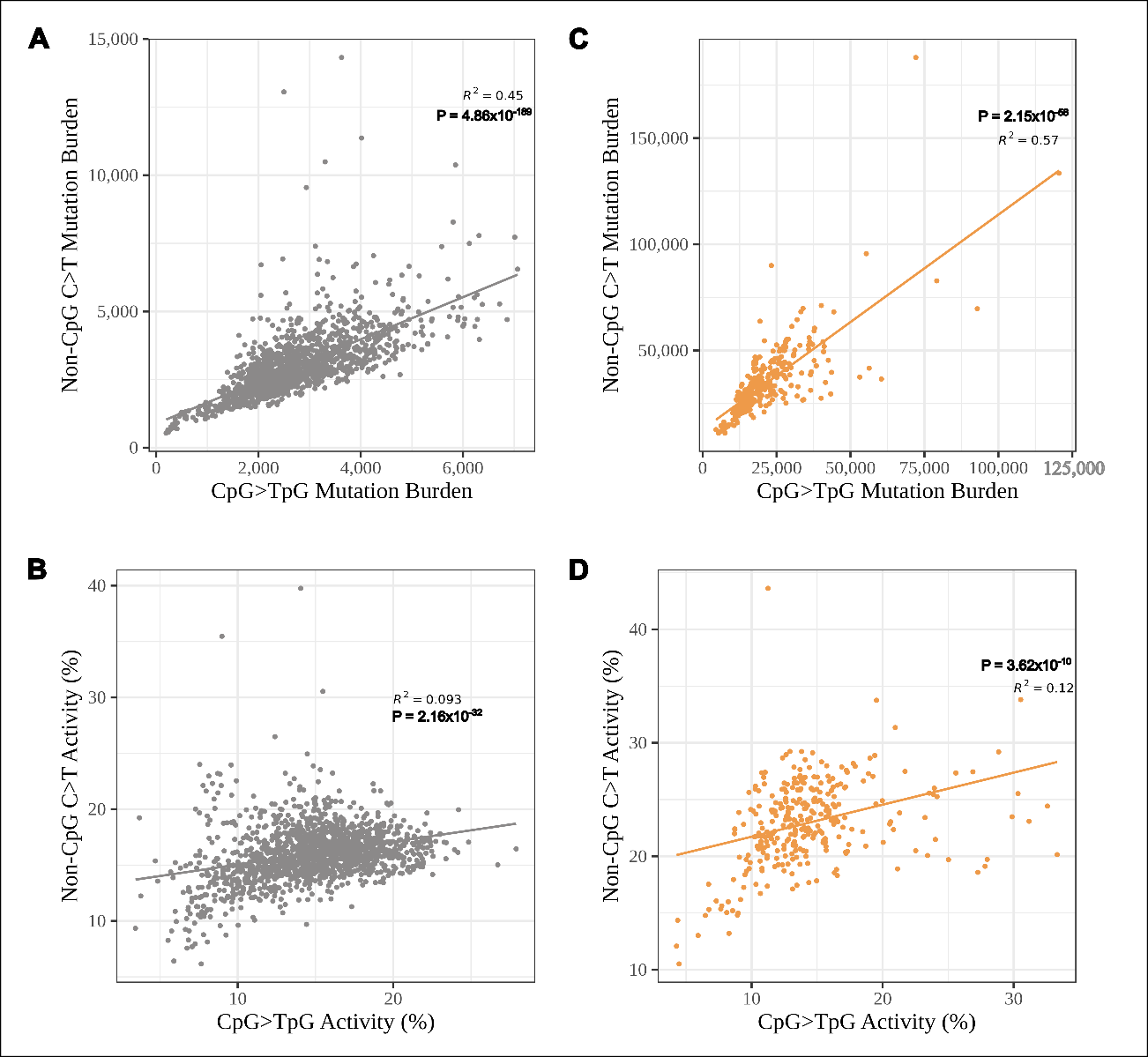
**Figure S2. Correlations between CpG>TpG and non-CpG C>T mutations in colorectal cancer.** Scatter plots showing the correlation between the CpG>TpG and non-CpG C>T burden and activity in mismatch repair-proficient (MMRp, grey; A-B) and mismatch repair-deficient (MMRd, orange; C-D) colorectal cancers.


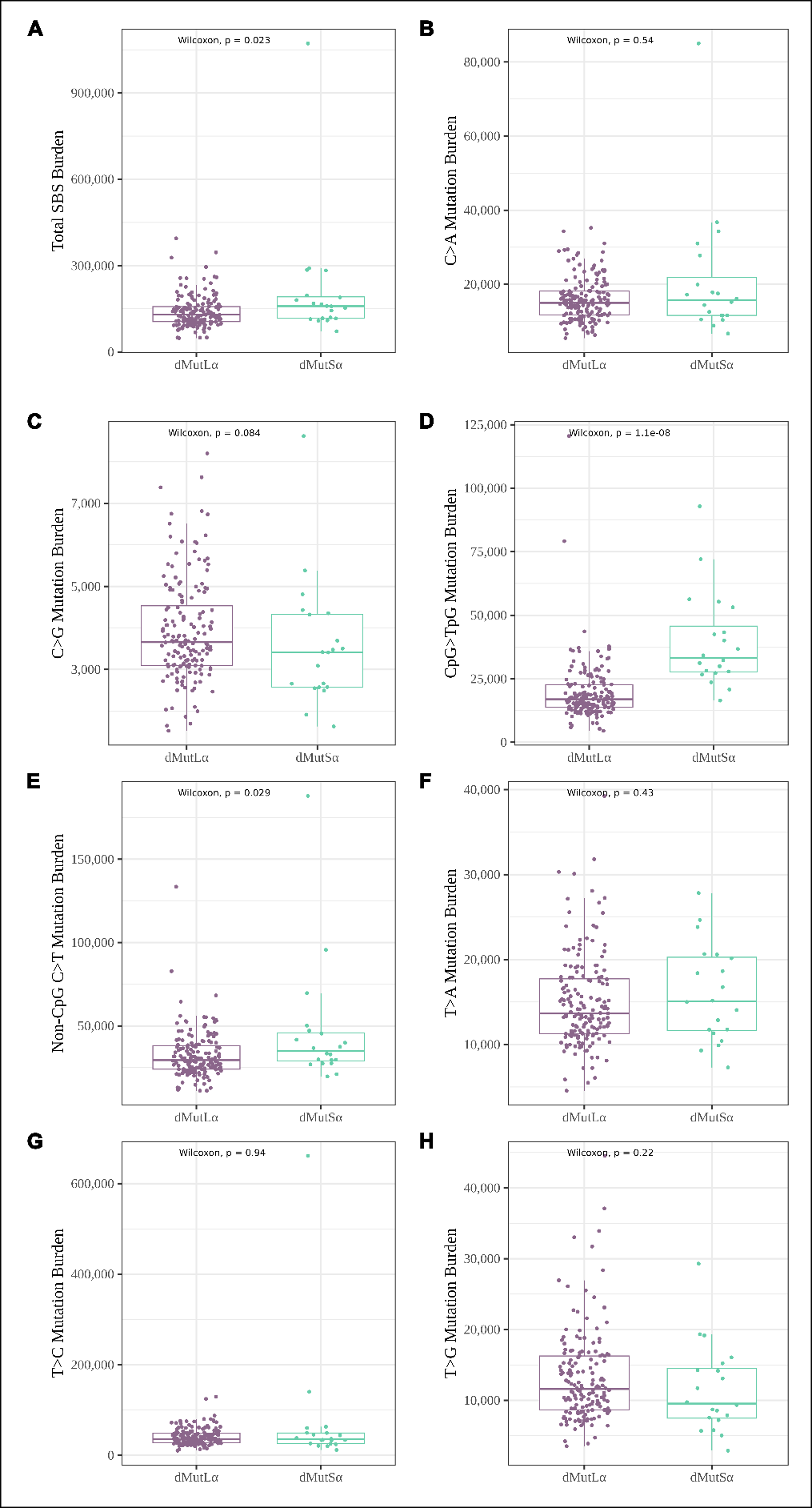
**Figure S3. The SBS mutation burden of MMRd colorectal cancers.** The total single-base substitution (SBS burden of mismatch repair-deficient (MMRd) colorectal cancers classified as either MutLα-deficient (dMutLα, purple) or MutSα-deficient (dMutSα, green) (A). Also shown are the burdens of the specific mutation channels C>A (B), C>G (C), CpG>TpG (D), Non-CpG C>T (E), T>A (F), T>C (G) and T>G (H).


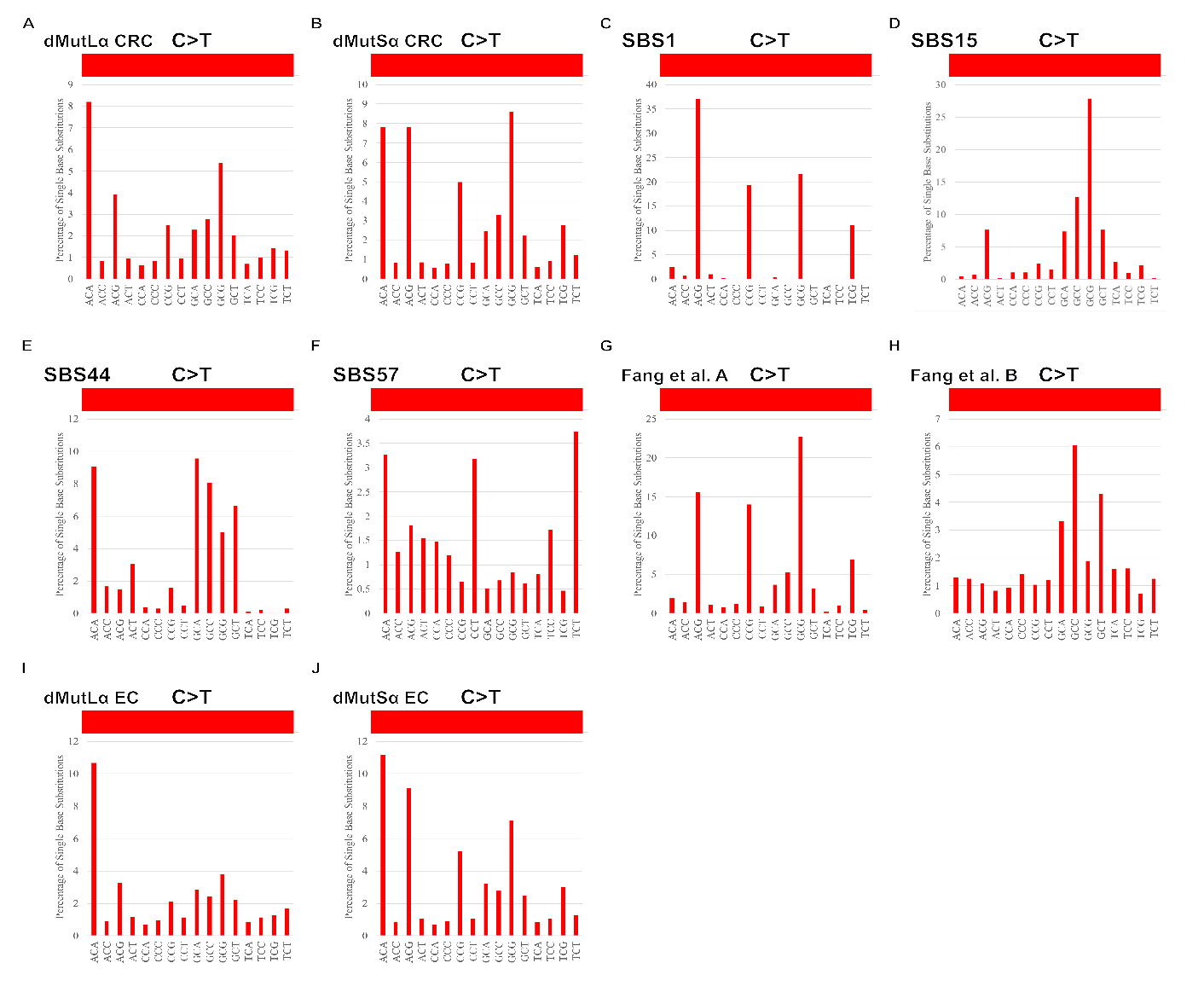


**Figure S4. C>T mutation channels of key mutation signatures.** The activities (%) sixteen C>T mutation channels in dMutLα and dMutSα colorectal cancer (A-B), as well as the reference signatures SBS1 (C), SBS15 (D), SBS44 (E), SBS57 (F) and the approximated *de novo* mutation signatures extracted from the study by Fang et al. (14) (G-H). Also shown are the dMutLα and dMutSα endometrial cancer spectra (I-J). COSMIC reference mutation signatures obtained from <https://cancer.sanger.ac.uk/signatures/>


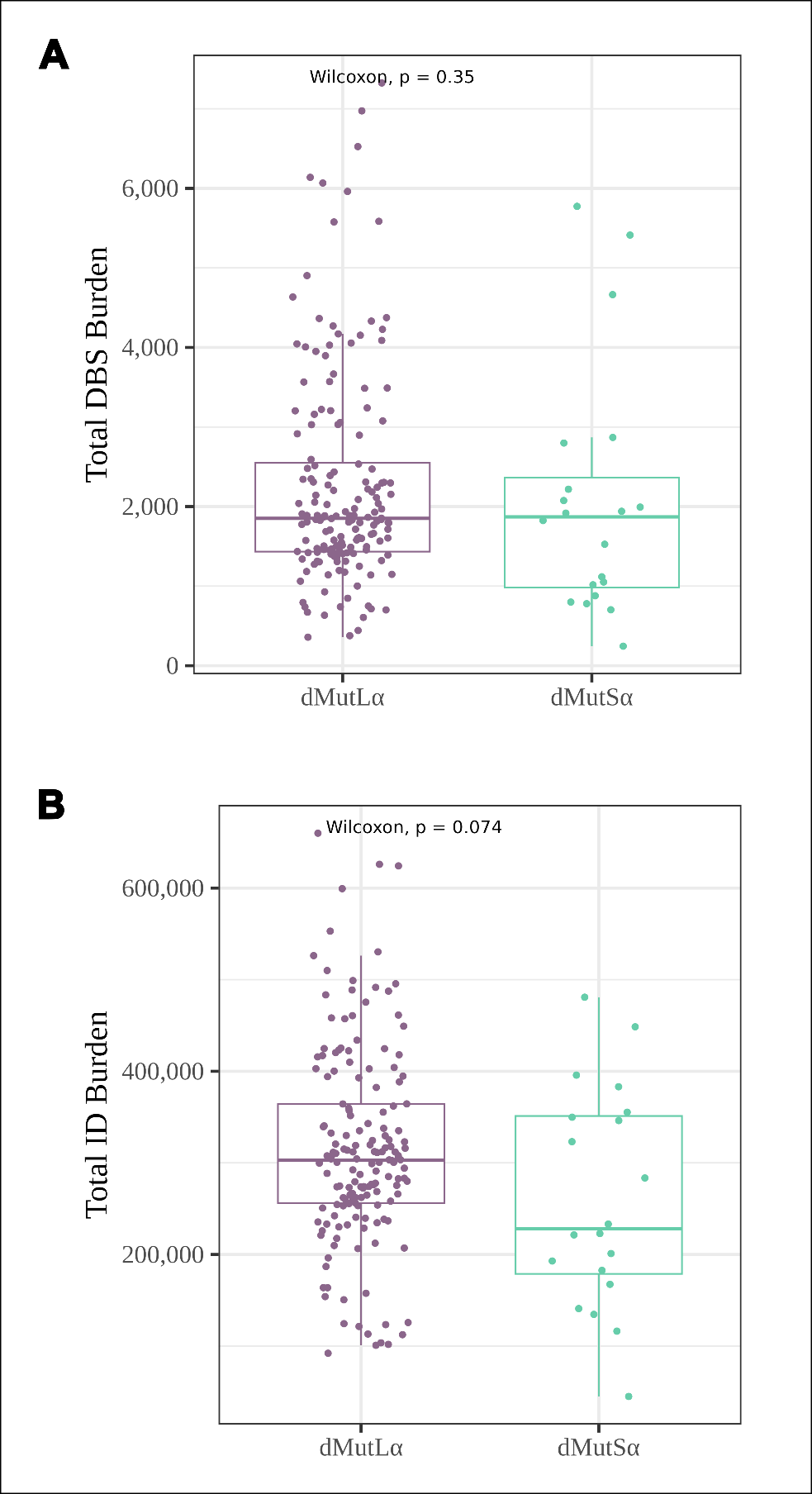
**Figure S5. DBS and ID mutations in MMRd colorectal cancers.** The total doublet-base substitution (DBS, A) and insertion-deletion (ID, B) mutation burden in mismatch repair deficient (MMRd) colorectal cancers classified as either MutLα-deficient (dMutLα, purple) or MutSα-deficient (dMutSα, green).

**
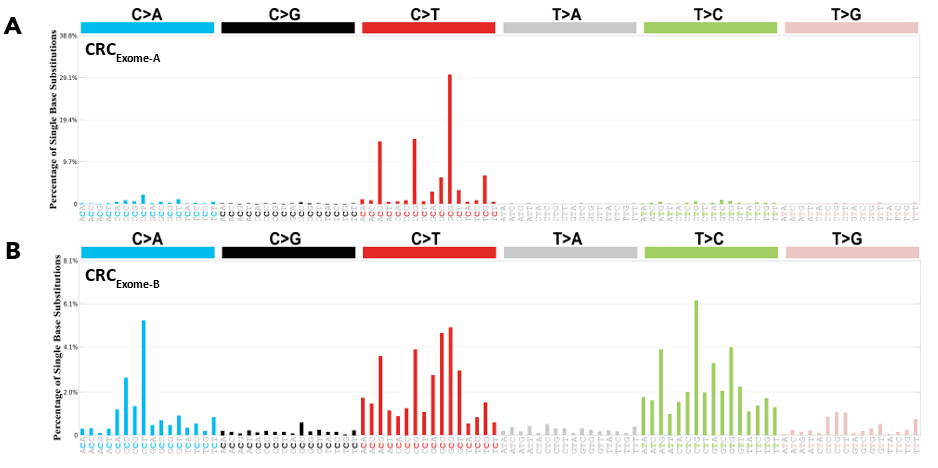
 Figure S6. *De novo* mutation signatures extracted from exonic DNA of MMRd CRCs.** Mutation signature plots of *de novo* mutation signatures CRC_Exome-A_ (A) and CRC_Exome-B_ (B), extracted from mutations within exonic DNA of mismatch repair-deficient (MMRd) colorectal cancers (CRCs).


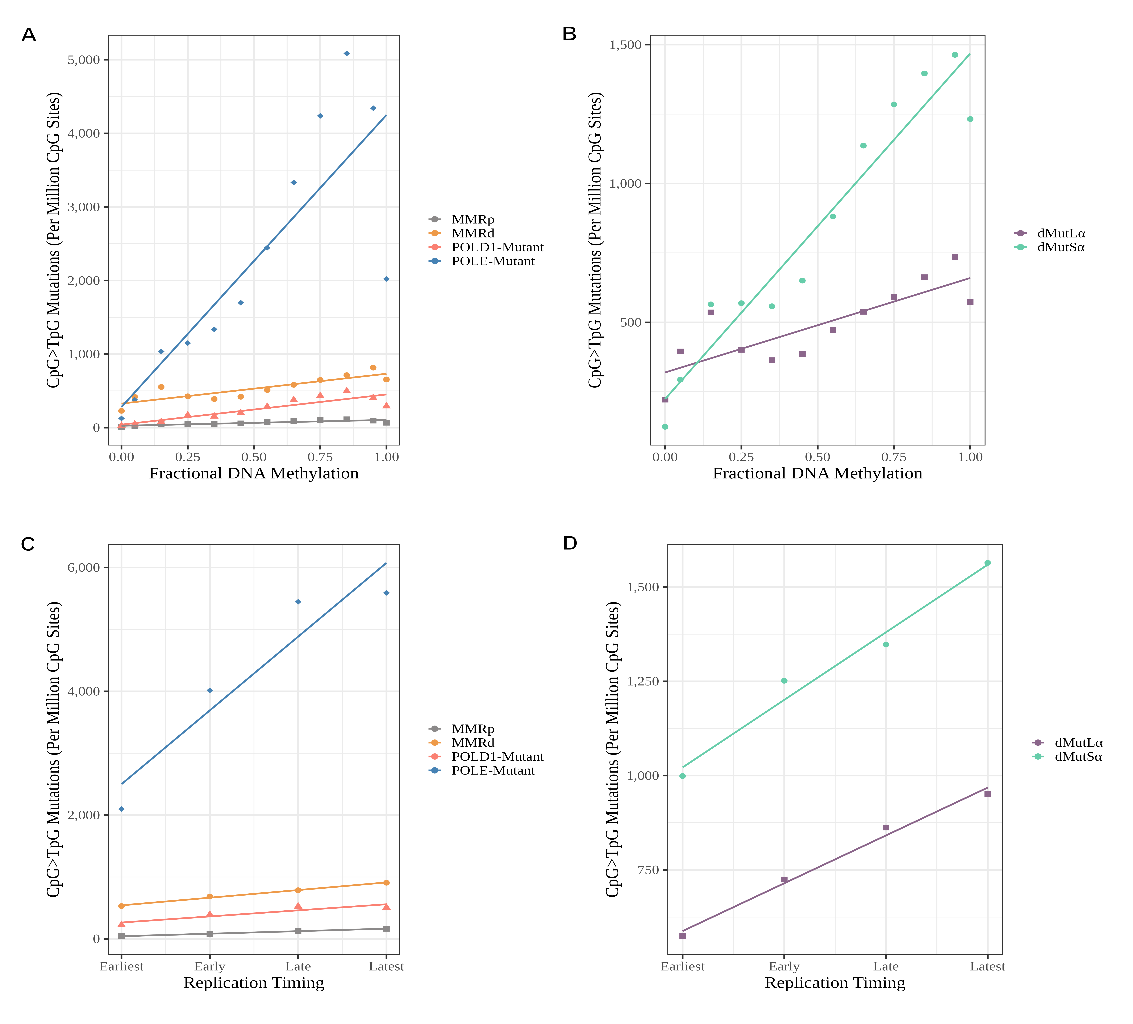
**Figure S7. CpG>TpG mutation association with DNA methylation & replication timing in colorectal cancer.** (A) The association between fractional DNA methylation and the CpG>TpG mutation rate (per million CpGs) in mismatch repair proficient (MMRp, grey), mismatch repair deficient (MMRd, orange), *POLE-*mutant (blue) and *POLD1-*mutant (red)colorectal cancers. (B) The association between fractional DNA methylation and the rate of CpG>TpG mutation in MMRd colorectal cancers classified as MutLα-deficient (dMutLα, purple) or MutSα-deficient (dMutSα, green). (C-D) The association between DNA replication timing and the CpG>TpG mutation rate (per million CpGs) for the same groups described in (A-B). Each data point represents the median value for each group in each DNA methylation or replication timing bin.

**Figure S8. Transcription strand bias of C>T mutations in MMRd CRC and EC.** The transcription strand (log_2_(Coding/Template)) bias of CpG>TpG and non-CpG C>T mutations in mismatch repair-deficient (MMRd) colorectal cancers (A) and endometrial cancers (B), classified as MutLα-deficient (dMutLα, purple) or MutSα-deficient (dMutSα, green). P comparing dMutLα to dMutSα from a Wilcoxon test. P comparing CpG>TpG *versus* non-CpG C>T from a Wilcoxon signed-rank test.


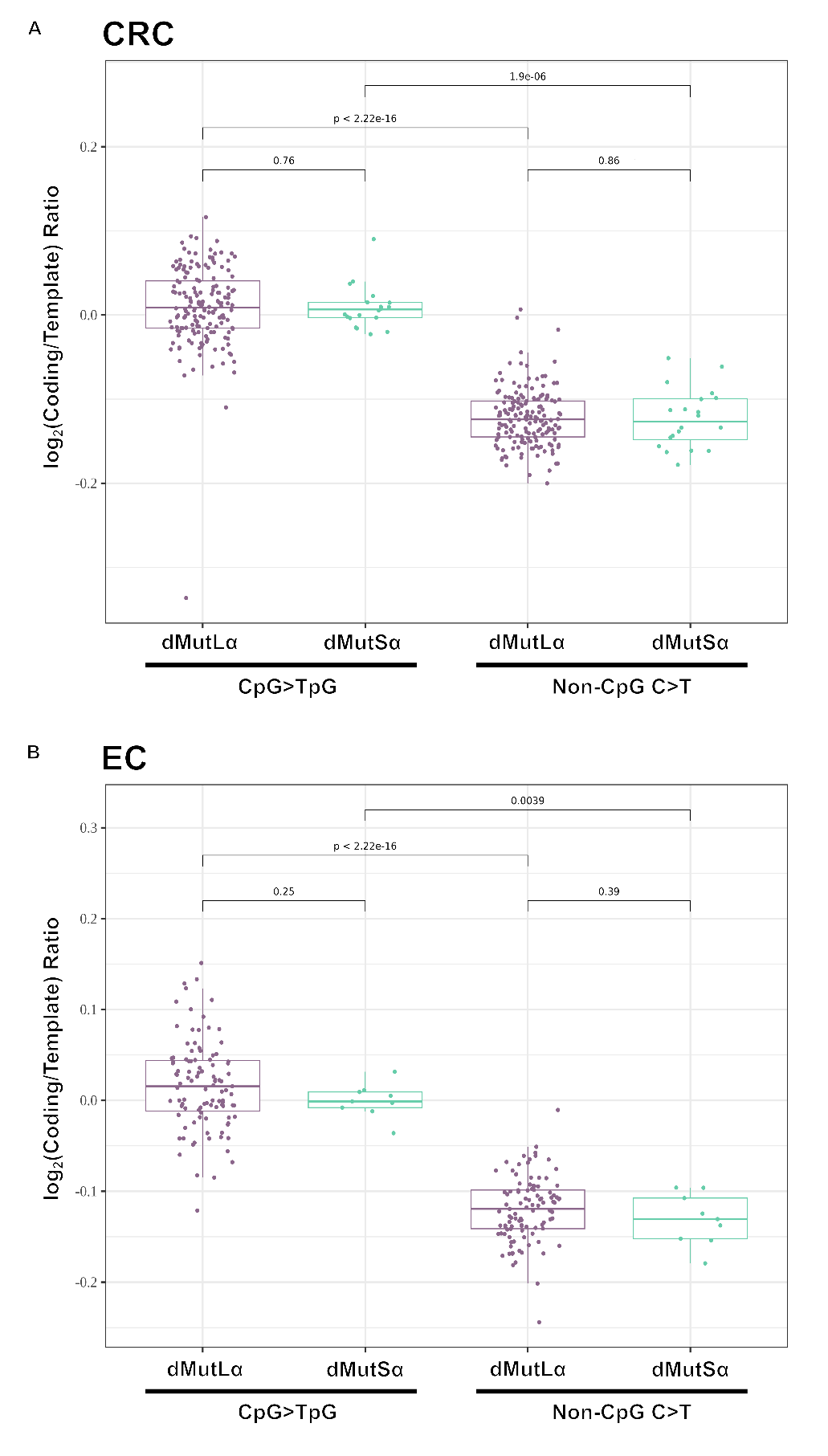


**Figure S9. Endometrial cancers included in mutation analysis.** A summary of the primary, treatment-naïve endometrial cancers (ECs) subjected to PCR-free whole-genome sequencing analysis from V18 of the UK 100,000 Genomes Project. mSINGS = Detecting MSI by Next-Generation Sequencing. MMRp=Mismatch repair-proficient. MMRd=Mismatch repair-deficient. POLE=DNA polymerase ε. POLD1=DNA polymerase δ.


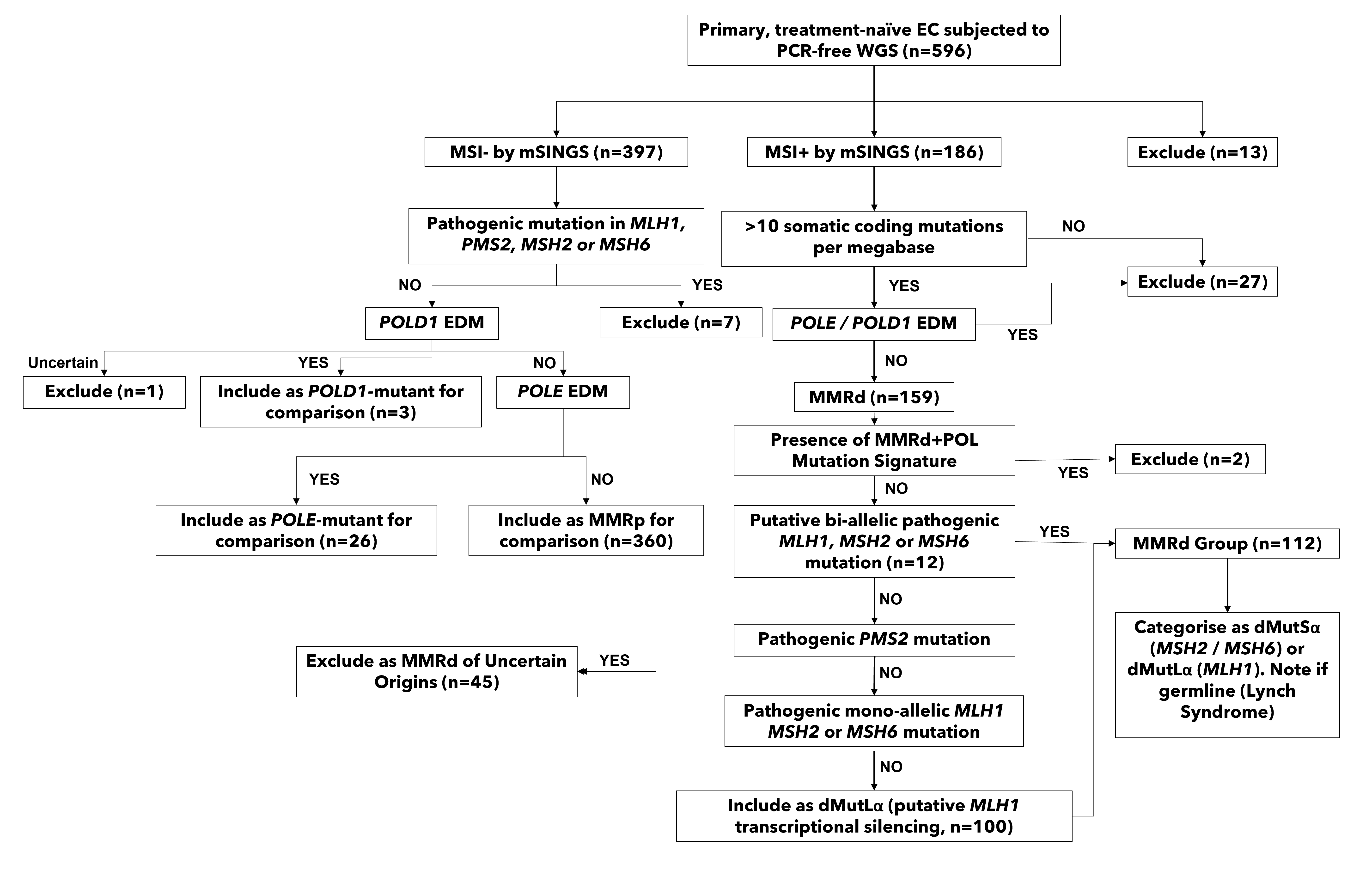


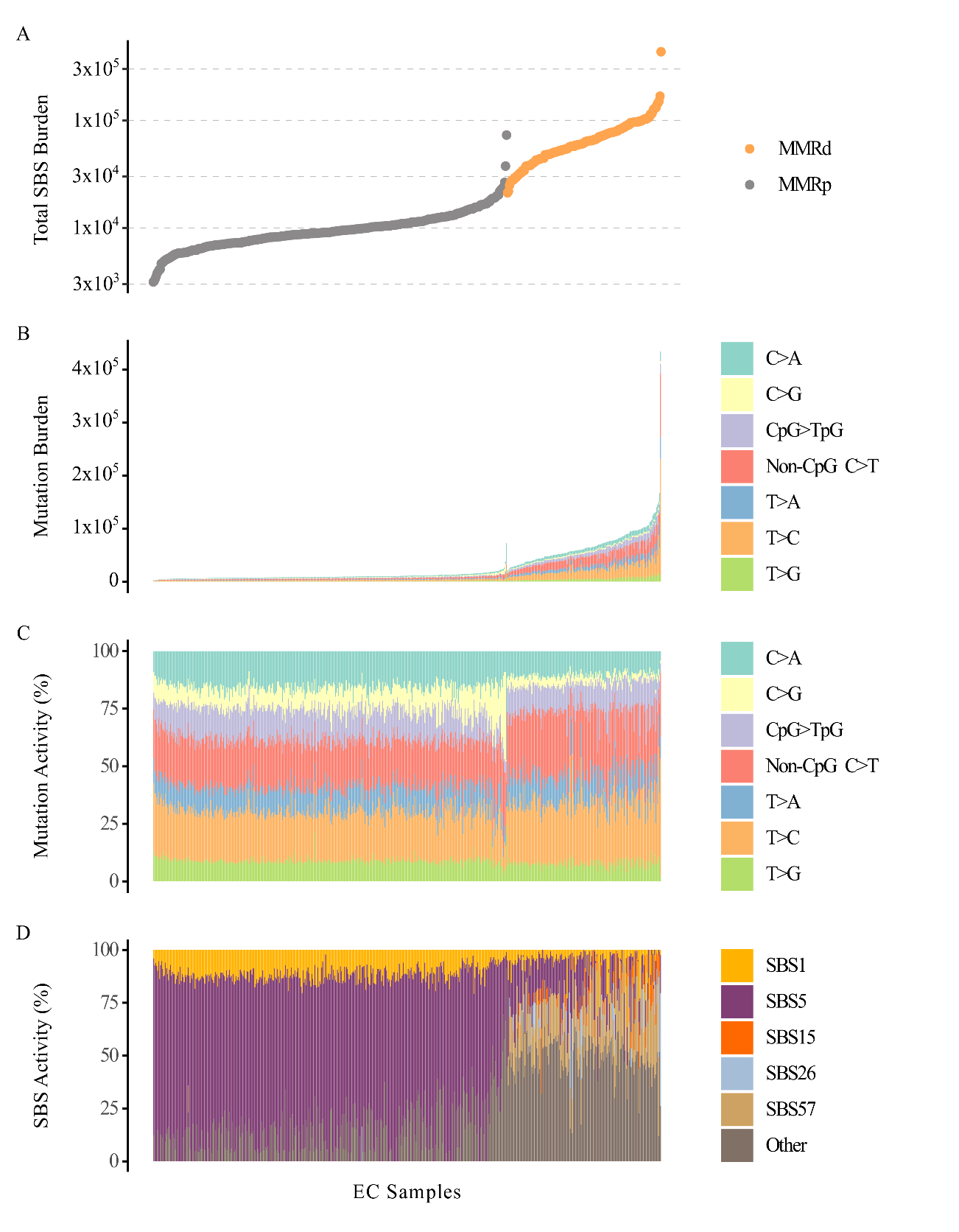
**Figure S10. The SBS landscapes of MMRp and MMRd endometrial cancers.** The total single base substitution (SBS) burden (A) of mismatch repair-proficient (MMRp, grey) and mismatch repair-deficient (MMRd, orange) endometrial cancers. Also shown are the burdens (B) and activities (C) of the six SBS mutation channels: C>A, C>G, C>T (split into CpG>TpG and non-CpG>TpG C>T mutations), T>A, T>C and T>G. (D) The activities of the “clock-like” SBS mutation signatures SBS1 and SBS5, as well as the MMRd-associated signatures SBS15, SBS26, SBS44 and the potential artefact signature SBS57 in the MMRp and MMRd endometrial cancers. Other mutations signatures include SBS2, SBS7c, SBS8, SBS10a, SBS10b, SBS10d, SBS13, SBS28, SBS51 and mutations not assigned to any pre-existing COSMIC mutation signature.


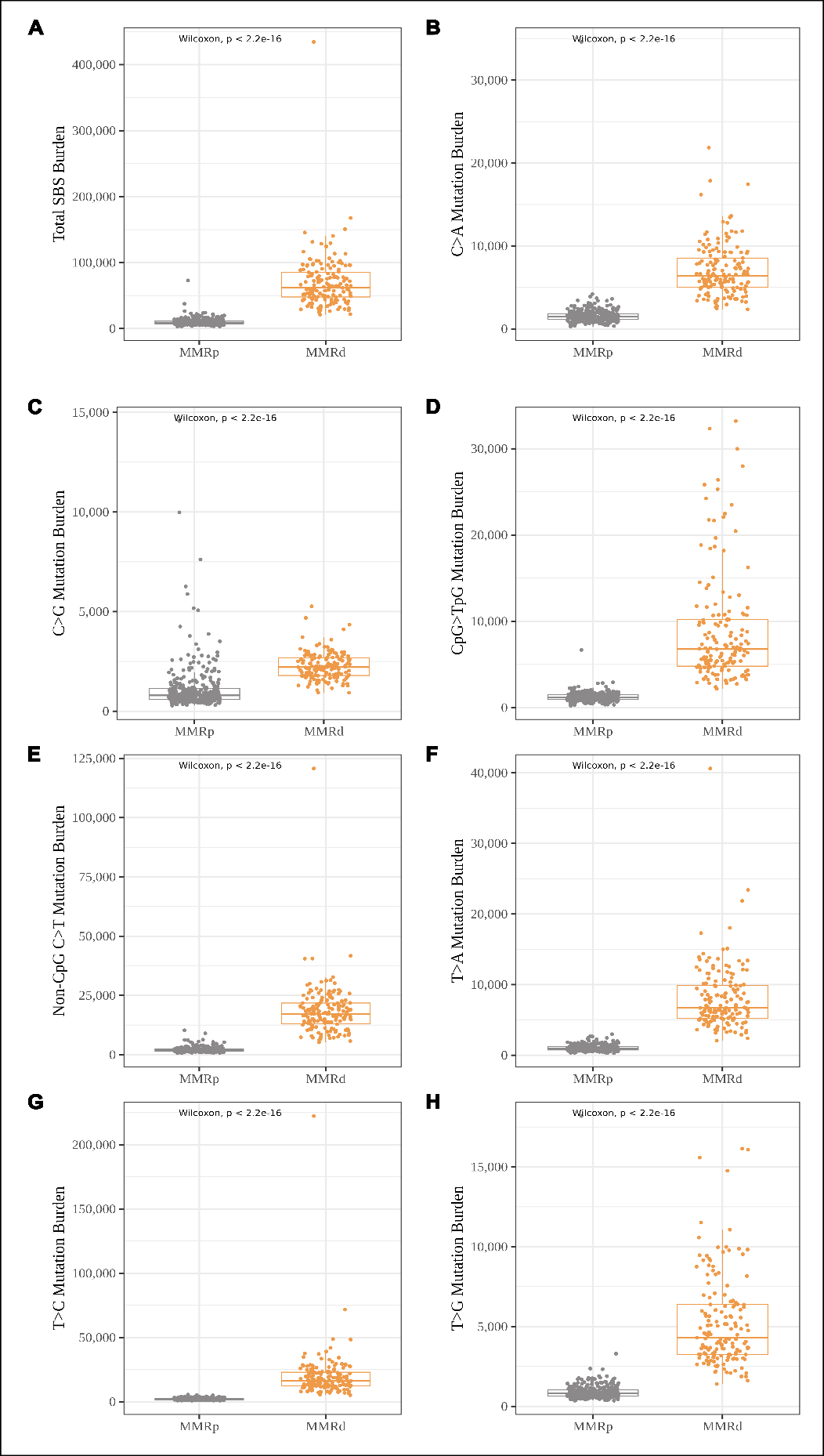
**Figure S11. The SBS mutation burden of MMRp and MMRd endometrial cancers.** The total single-base substitution (SBS burden of mismatch repair-proficient (MMRp, grey) and mismatch repair-deficient (MMRd, orange) endometrial cancers (A). Also shown are the burdens of the specific mutation channels C>A (B), C>G (C), CpG>TpG (D), Non-CpG C>T (E), T>A (F), T>C (G) and T>G (H).


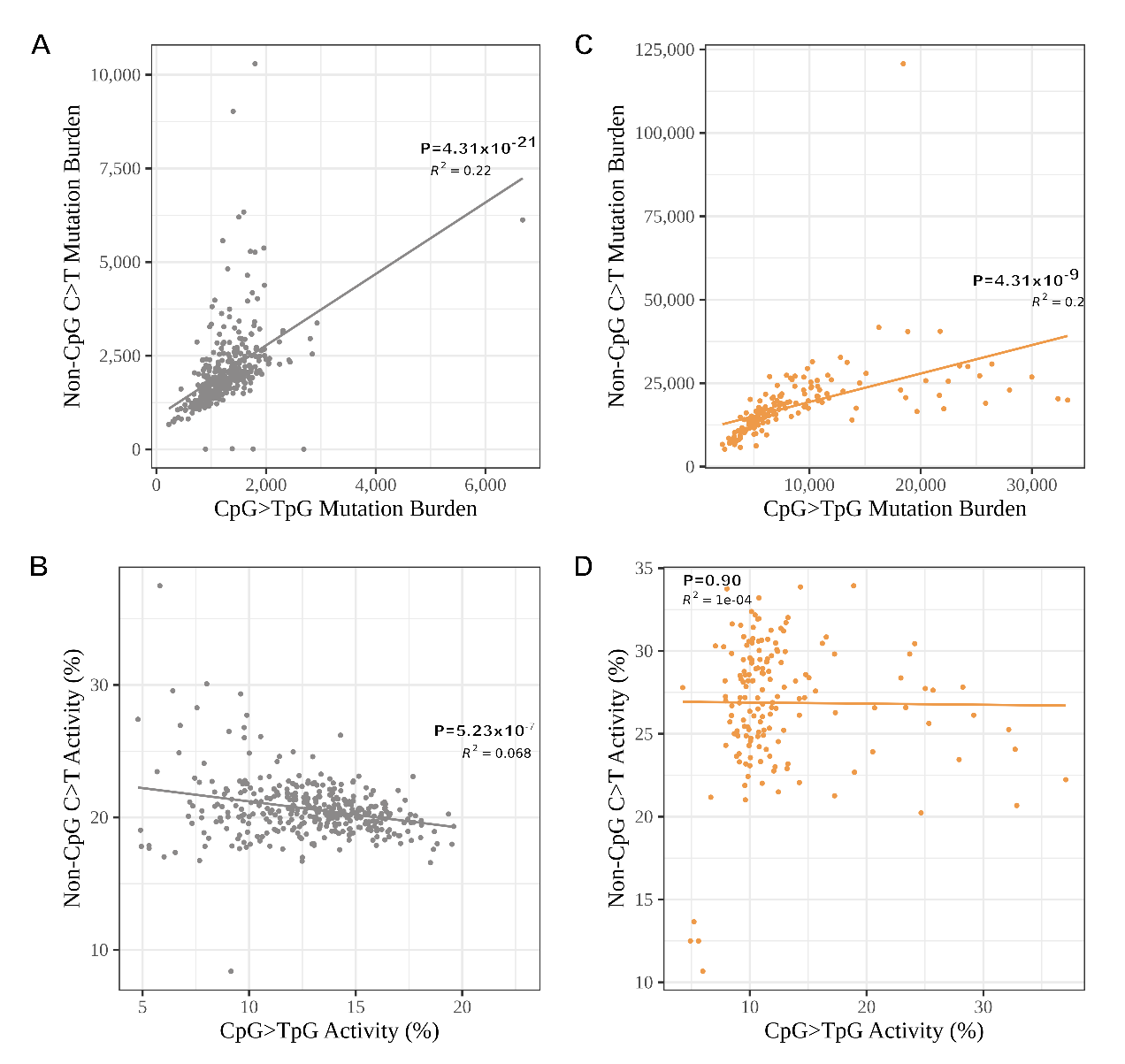
**Figure S12. Correlations between CpG>TpG and non-CpG C>T mutations in endometrial cancer.** Scatter plots showing the correlation between the CpG>TpG and non-CpG C>T burden and activity in mismatch repair-proficient (MMRp, grey; A-B) and mismatch repair-deficient (MMRd, orange; C-D) endometrial cancers.


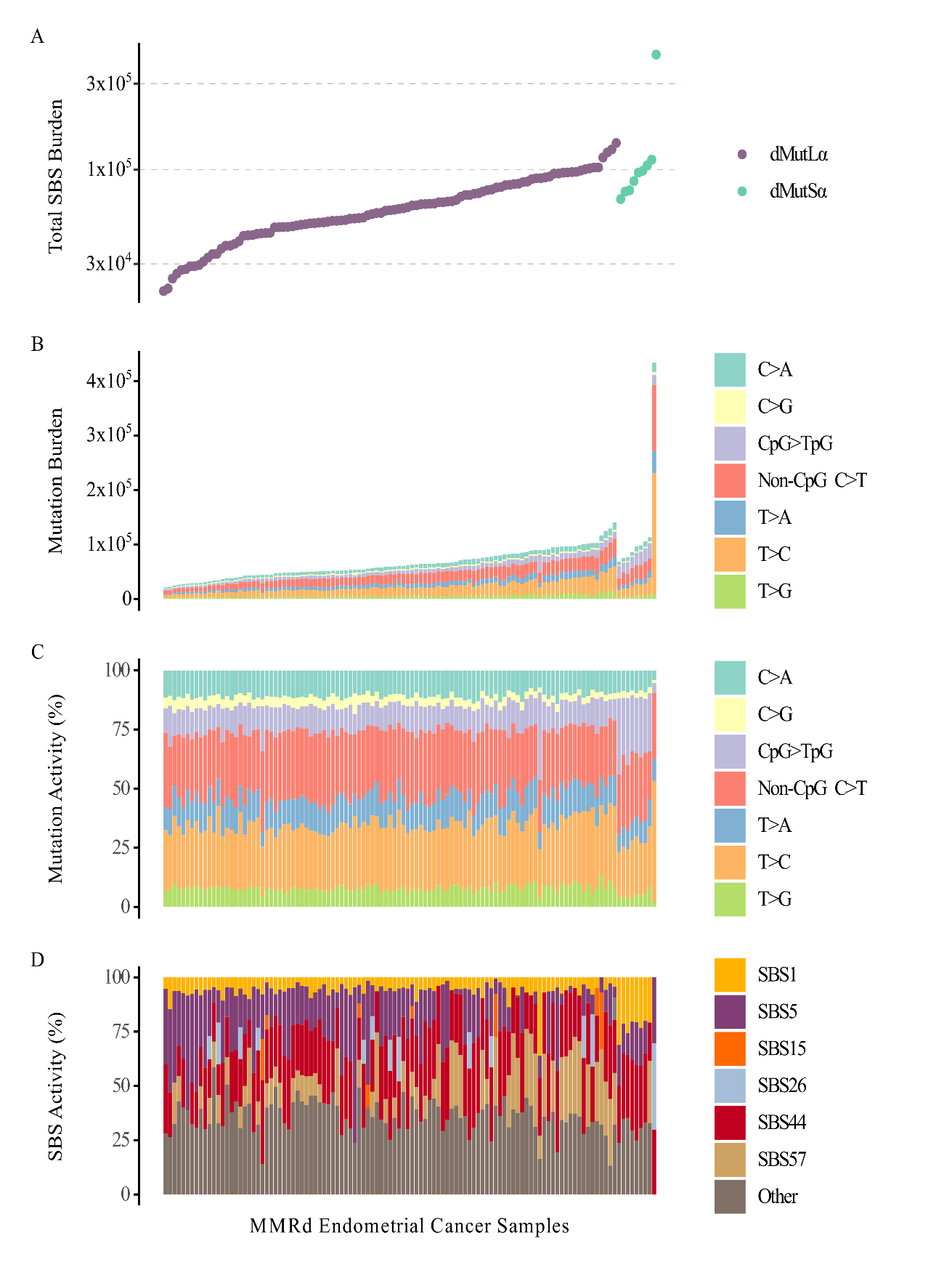
**Figure S13. The SBS landscapes of dMutS*α* and dMutL*α* endometrial cancers.** The total single-base substitution (SBS) burden (A) of MutLα-deficient (dMutLα, purple) or MutSα-deficient (dMutSα, green) endometrial cancers. Also shown are the burdens (B) and activities (C) of the six SBS mutation channels: C>A, C>G, C>T (split into CpG>TpG and non-CpG C>T mutations), T>A, T>C and T>G. (D) The activities of the “clock-like” SBS mutation signatures SBS1 and SBS5, as well as the MMRd-associated signatures SBS15, SBS21, SBS26, SBS44 and the potential artefact signature SBS57 in the dMutLα and dMutSα endometrial cancers. Other mutation signatures include SBS7c, SBS9 and mutations not assigned to any pre-existing COSMIC mutation signature.

**Figure S14. The SBS mutation burden of MMRd endometrial cancers.** The total single-base substitution (SBS burden of mismatch repair-deficient (MMRd) endometrial cancers classified as either MutLα-deficient (dMutLα, purple) or MutSα-deficient (dMutSα, green) (A). Also shown are the burdens of the specific mutation channels C>A (B), C>G (C), CpG>TpG (D), Non-CpG C>T (E), T>A (F), T>C (G) and T>G (H).


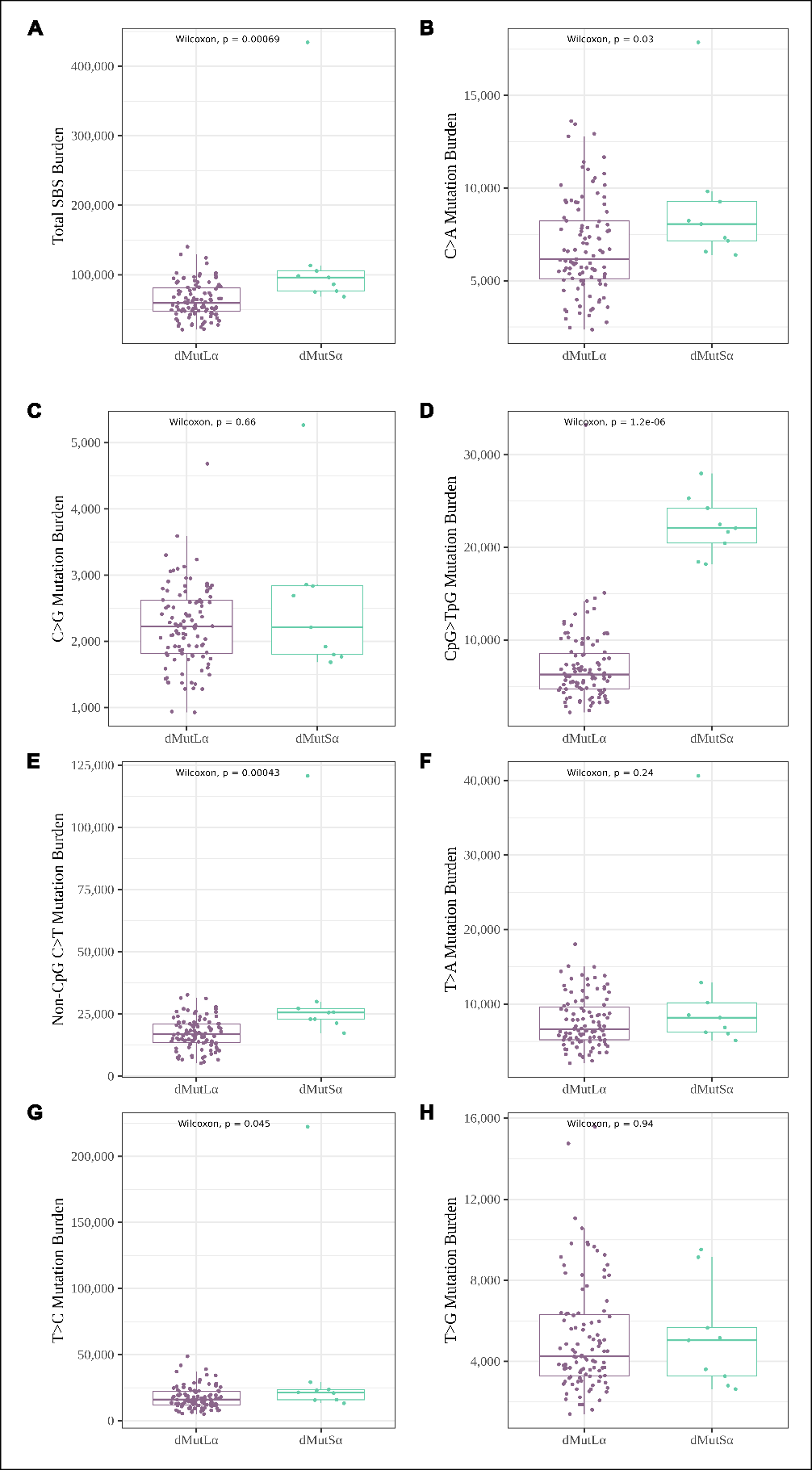


**Figure S15. DBS and ID mutations in MMRd endometrial cancers.** The total doublet-base substitution (DBS, A) and insertion-deletion (ID, B) mutation burden in mismatch repair deficient (MMRd) endometrial cancers classified as either MutLα-deficient (dMutLα, purple) or MutSα-deficient (dMutSα, green)


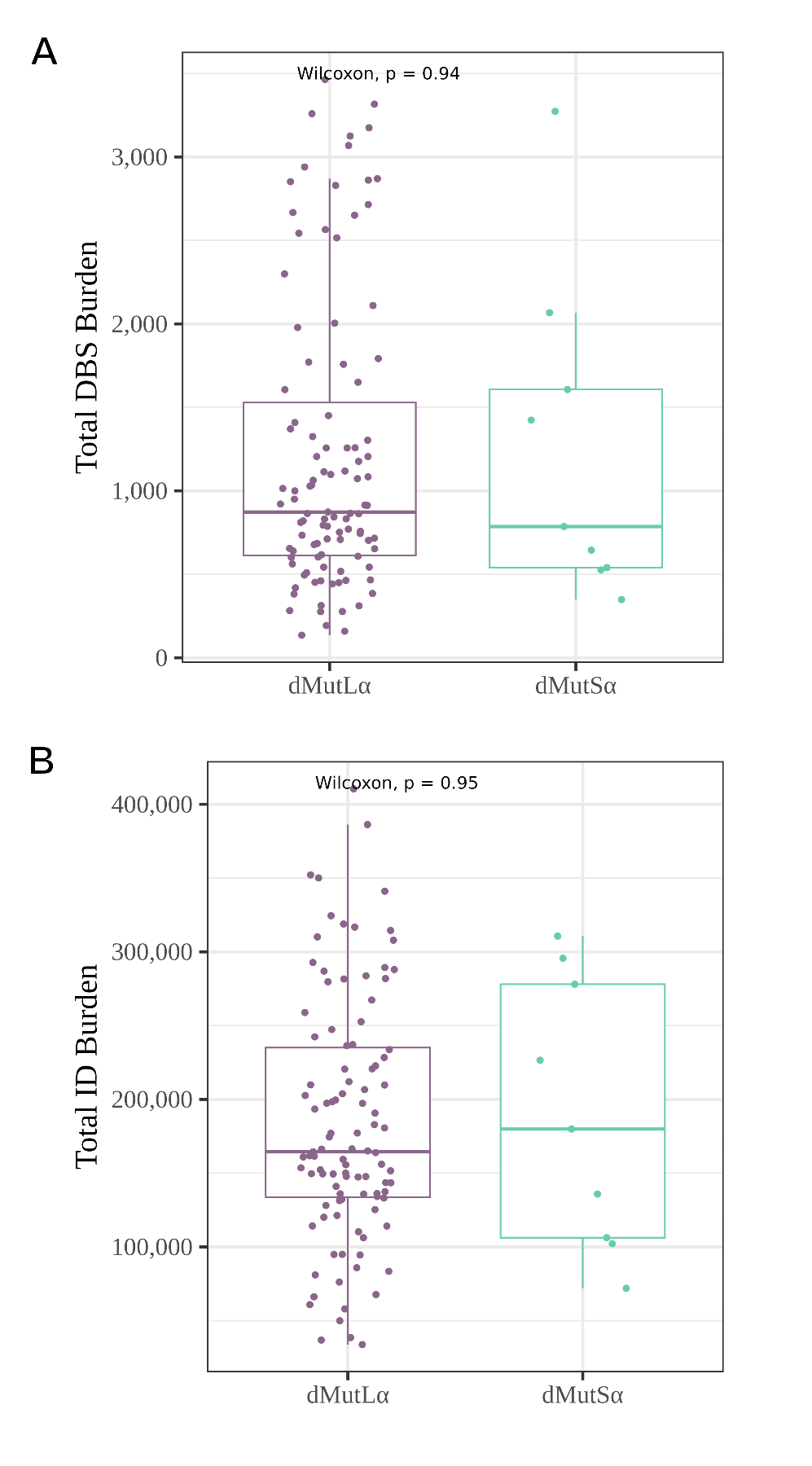


**Figure S16. Mutation spectra of dMutLα and dMutSα endometrial cancers cancers.** The activities (proportions of all 96 SBS channels) of each specific substitution are shown for dMutLα ECs (A), dMutSα ECs (B) and the *de novo* mutation signatures SBS_EC-MMRd-A_ (C) and SBS_EC-MMRd-B_ (D) Also shown are the pairwise cosine similarities of the sixteen C>T mutation channels of these dMutLα and dMutSα endometrial cancers with the *de novo* mutation signatures, the COSMIC signatures SBS1, SBS15, SBS44 and SBS57 and the *de novo* mutation signatures Signature A and Signature B from the study by Fang et al. (14) (E).


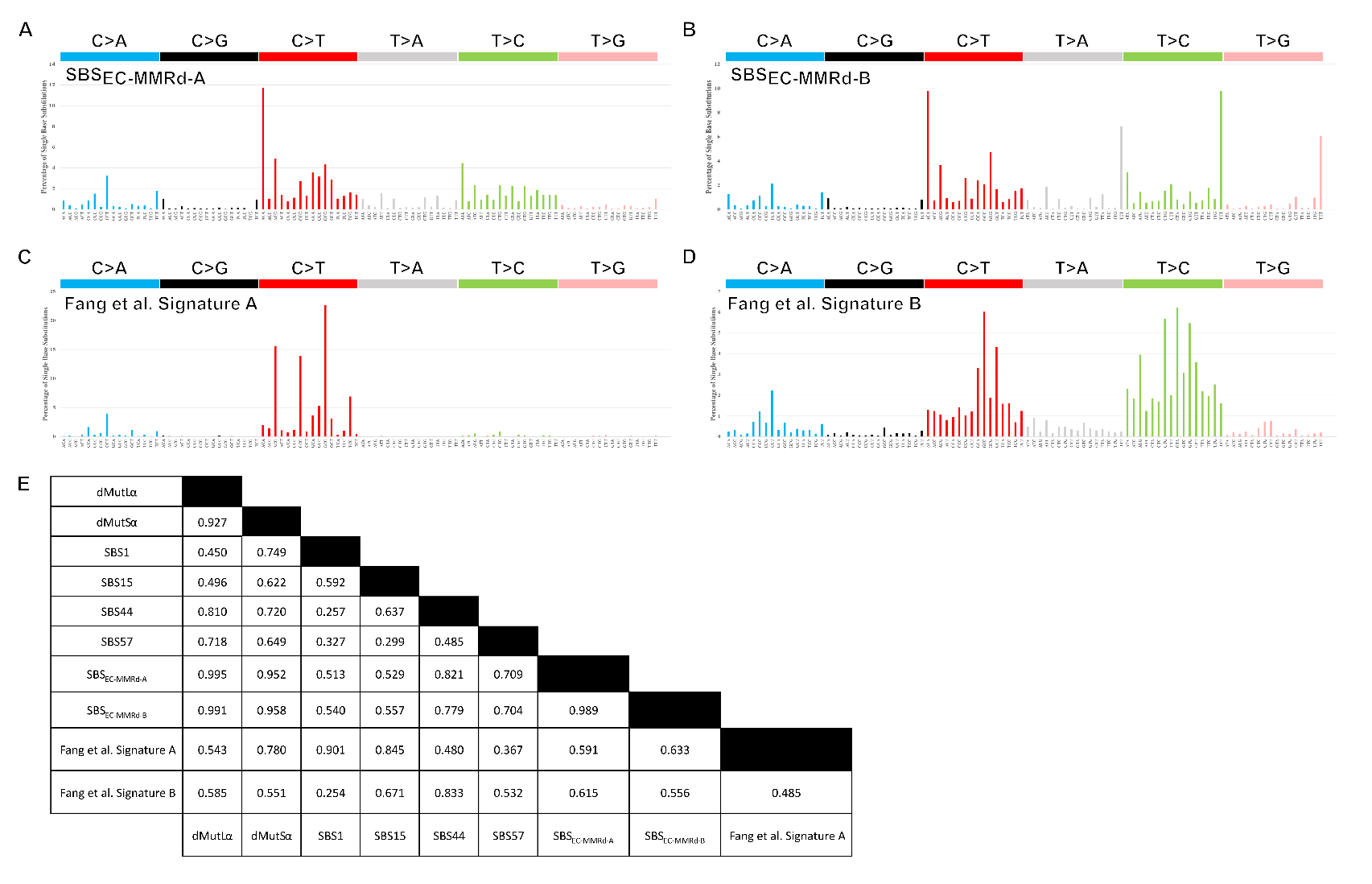


**Figure S17. Addition of CpG>TpG mutations in proportion to SBS1 channel activities to the observed dMutLα mutation spectrum causes near-identity to the observed dMutSα spectrum in endometrial cancer.** The y-axis shows the cosine similarity between CpG>TpG mutation spectrum of dMutSα CRCs and the same spectrum in dMutLα CRCs when SBS1-associated mutations are proportionally added. As more SBS1 mutations are added (*i.e.* x-axis values increase), the cosine similarity rises to a peak of 0.972. The effects of adding in SBS15 and SBS44 channels are shown for comparison, consistent with a small possible additional effect of both signatures.


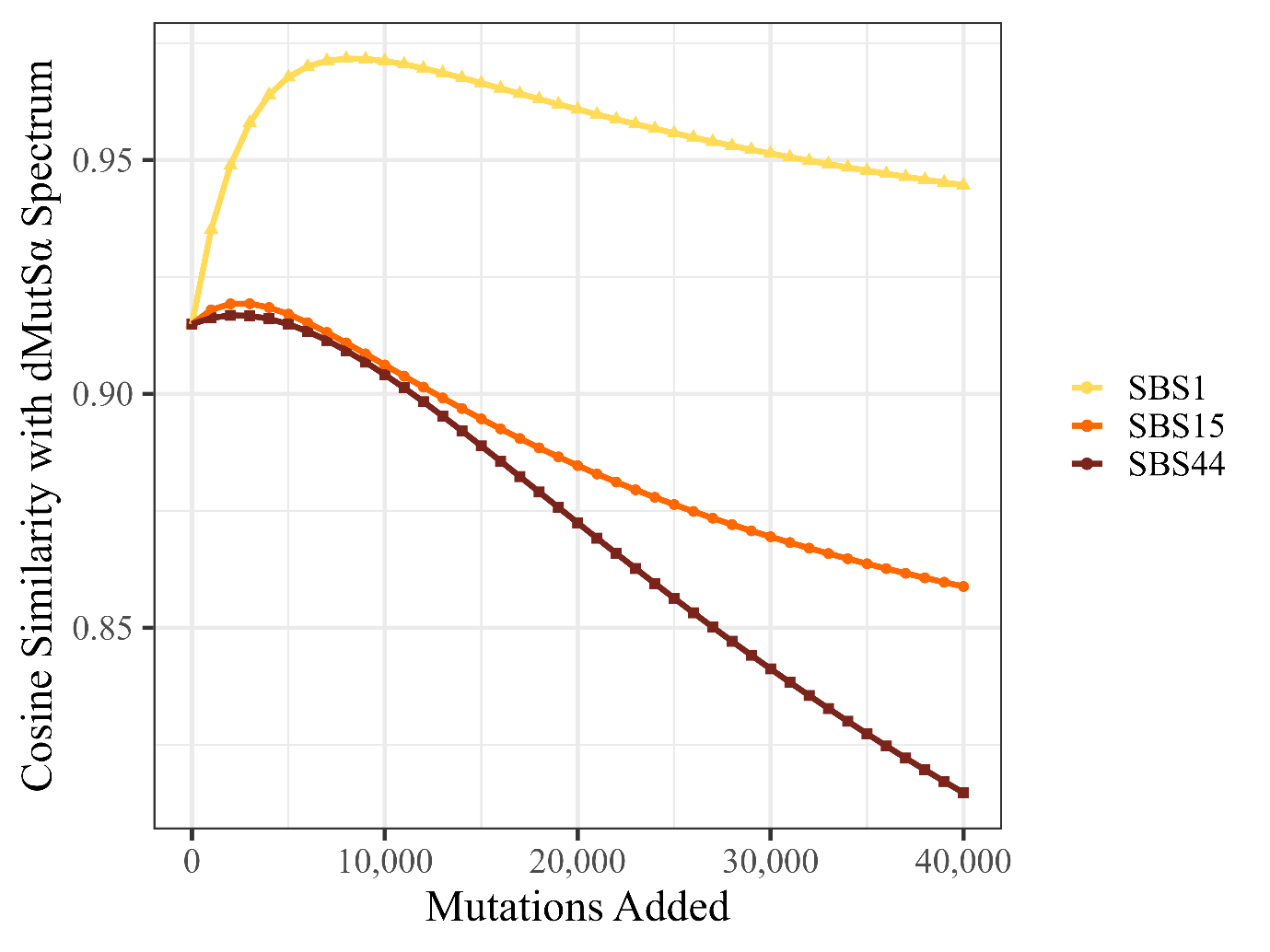


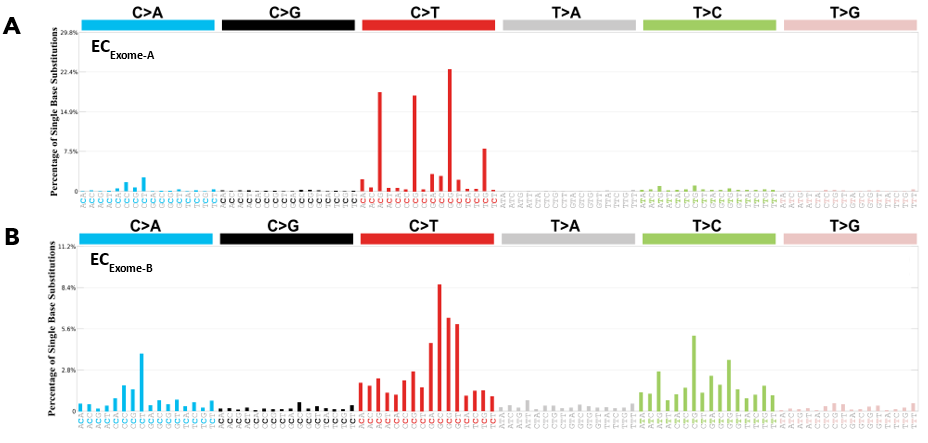
**Figure S18. *De novo* mutation signatures extracted from exonic DNA of MMRd ECs.** Mutation signature plots of *de novo* mutation signatures EC_Exome-A_ (A) and EC_Exome-B_ (B), extracted from mutations within exonic DNA of mismatch repair-deficient (MMRd) endometrial cancers (ECs).
