# Supplementary material for: Replication-associated mechanisms contribute to an increased CpG > TpG mutation burden in mismatch repair-deficient cancers
Source: Genome Med. 2025 Aug 25;17:95. doi: 10.1186/s13073-025-01525-6 (PMC12376743; doi:10.1186/s13073-025-01525-6)
Supplement: Supplementary file 3 — Additional file 3: Ward_et_al_Additional_File_3.docx. Analysis of 100kGP endometrial cancers [file 13073_2025_1525_MOESM3_ESM.docx]

**Additional File 3: Analysis of C>T mutations in endometrial cancers (ECs)**

Mutational features of MMRp and MMRd ECs are shown in **Additional File 2** (**Figures S10** & **S11**). We observed a positive correlation between CpG>TpG and non-CpG C>T mutation burdens, and a negative correlation for activity, in MMRp ECs (**Additional File 2: Figure S12A-B**), whereas a positive correlation between CpG>TpG and non-CpG C>T burdens was observed in MMRd ECs, with no correlation present between the activities (**Additional File 2: Figure S12C-D**). Also, similar to CRCs, we found minimal differences in the molecular features of *MLH1-*mutant *versus* putative *MLH1* promoter hypermethylation, *MSH2- versus* *MSH6-*mutant and germline *versus* somatic dMutLα and dMutSα ECs (**Additional File 1:** **Tables S24-S27**). Therefore, we again combined the sub-types of MMRd EC into dMutSα (n=9) and dMutLα (n=103) groups, in the same way as for CRC.

The comparative analyses of dMutSα and dMutLα ECs are shown in **Additional File 1** (**Table S28**) and **Additional File 2** (**Figures S13** & **S14**). We found that dMutSα ECs had a significantly greater SBS burden than dMutLα tumours (median 96,264 *versus* 59,497; *P*=6.9x10^-4^). Consistent with what was observed in CRCs, dMutSα ECs had a significantly greater CpG>TpG and non-CpG C>T burden than dMutLα ECs (median 22,079 *versus* 6,274; P=1.2x10^-6^ for CpG>TpG and median 25,594 *versus* 16,943; P=4.3x10^-4^ for non-CpG C>T). Interestingly, the C>A and T>C mutation burdens were also increased in dMutSα ECs (median 8,057 *versus* 6,161; P=0.03 for C>A and median 21,481 *versus* 16,114; P=0.045 for T>C), while there was no difference in the C>G, T>A and T>G burdens between these two MMRd sub-types.

Furthermore, while the activities of five of the six SBS types were greater in dMutLα ECs than dMutSα, the activity of CpG>TpG mutations was greater in dMutSα (median 24.1% *versus* 10.5%; P=1.6x10^-4^), while there was no difference in the activity of non-CpG C>T changes (median 27.8% *versus* 27.6%; P=0.91). There were minimal differences in the prevalence of C>T-associated mutation signatures in dMutLα and dMutSα ECs (**Additional File 1: Table S29**), with SBS44 being the most prevalent MMRd-associated signature. Interestingly, SBS57 was more prevalent in dMutLα ECs than dMutSα (present in 58.3% *versus* 11.1%; P=0.011; Fisher’s exact test; **Additional File 1:** **Table S29**). The activity of SBS1 was significantly higher in dMutSα ECs (median 20.9% *versus* 5.4%; *P*=1.6x10^-4^). The only other difference in SBS mutation signatures was higher SBS57 activity in dMutLα ECs (median 10.2% *versus* 0% *P*=0.02). The activity of all other mutation signatures not specifically presented in **Additional File 2** (**Figure S13**) was also greater in dMutLα ECs (median 56.4% *versus* 30.6%; P=0.007).

Comparisons between dMutSα ECs and *MLH1-*mutant ECs – that is, cancers for which the dMutSα *versus* dMutLα classification was not assigned by exclusion – were relatively underpowered, with only nine dMutSα ECs and three dMutLα ECs. Nevertheless, there was a significantly increased CpG>TpG burden in the former (*P=*0.0091; **Additional File 1:** **Table S12**).

There were no differences in the DBS (median 873 *versus* 786; P=0.94; **Additional File 1:** **Table S30**) and ID (median 164,492 *versus* 179,858; P=0.95; **Additional File 1:** **Table S31**) burdens and signatures between dMutLα and dMutSα ECs (**Additional File 2: Figure S15**). In dMutSα ECs, the major SBS1 channels – ACG>ATG, GCG>GTG, CCG>CTG and TCG>TTG – showed smaller deviations from SBS1 than were observed in CRC (P=2.0x10^-10^ for both dMutSα and dMutLα; χ^2^ test), with GCG>GTG mutations comprising 35.9% and 30.7% of CpG>TpG mutations in dMutLα and dMutSα ECs respectively (**Additional File 1:** **Table S32**). Of the other CpG>TpG changes in dMutLα dMutSα ECs, the next most common were ACG>ATG (32% and 36% respectively), followed by CCG>CTG (20.3% and 21.2% respectively) and finally TCG>TTG (11.8% and 12.1% respectively) mutations.

As in CRCs, the cosine similarity between the dMutSα and dMutLα mutation spectra was high (0.915 for all 96 SBS channels, 0.927 for the 16 C>T channels only; **Additional File 2: Figure S16A-B**). As in CRC, the similarity between the four CpG>TpG mutation channels in dMutLα and dMutSα ECs was increased by the addition of SBS1-associated mutations to the former, raising the cosine similarity to a maximum of 0.972 (**Additional File 2: Figure S17**). Unlike in CRC, the addition of SBS15- and SBS44-associated CpG>TpG mutations lead to a small increase in cosine similarity (peaking at 0.919 and 0.917 respectively; **Additional File 2: Figure S17**).

*De novo* SBS mutation signature extraction was performed in MMRd ECs, again setting the number of novel signatures to two (SBS_EC-MMRd-A_ and SBS_EC-MMRd-B_; cosine similarity 0.786 for the ninety-six SBS mutation channels and cosine similarity 0.989 for the sixteen C>T mutation channels; **Additional File 1: Table S33**; **Additional File 2:** **Figure S16C-E**;). There were no differences in the prevalences of SBS_EC-MMRd-A_ and SBS_EC-MMRd-B_ between dMutLα and dMutSα ECs (SBS_EC-MMRd-A_ present in 96.1% *versus* 100%; P>0.99; Fisher’s exact test and SBS_EC-MMRd-B_ present in 100% of dMutLα and dMutSα ECs). The activity of C>T mutations in SBS_EC-MMRd-A_ and SBS_EC-MMRd-B_ was 44.4% and 35.5% respectively, with CpG>TpG activities of 13.6% and 12.5% respectively. Both *de novo* signatures were characterised by ACA>ATA mutations, similar to the signatures extracted from MMRd CRCs. The sixteen C>T mutation channels in SBS_EC-MMRd-A_ and SBS_EC-MMRd-B_ were most similar to the COSMIC signature SBS44 (cosine similarities 0.821 and 0.779 respectively; **Additional File 2: Figure S16E**). Interestingly, while ACG>ATG was the most common CpG>TpG mutation in SBS_EC-MMRd-A_, the sixteen C>T mutation channels in SBS_EC-MMRd-B_ was more similar to SBS1 than SBS_EC-MMRd-A_ (cosine similarities 0.540 and 0.513 respectively; **Additional File 2: Figure S16E**). Furthermore, the sixteen C>T mutation channels in SBS_EC-MMRd-A_ had a lower cosine similarity with SBS1 than SBS_CRC-MMRd-A_ (0.513 *versus* 0.756). The sixteen C>T mutation channels in dMutSα ECs more closely resembled Fang et al. (14) Signature A (cosine similarity 0.780; **Additional File 2: Figure S16E**), while dMutLα more closely resembled signature B (cosine similarity 0.585). SBS_EC-MMRd-B_ was characterised by ACA>ATA, GCN>GTN and TTT>TCT mutations, similar to SBS_CRC-MMRd-B_ (sixteen C>T mutation channel cosine similarity 0.938). Overall, the activity of SBS_EC-MMRd-A_ was significantly greater in dMutSα ECs (median 81% *versus* 62.8%; P=2.1x10^-4^), while the activity of SBS_EC-MMRd-B_ was significantly greater in dMutLα tumours (median 37.2% *versus* 19%; P=2.1x10^-4^).

We then extracted two *de novo* mutation signatures from exonic regions of MMRd ECs, termed EC_Exome-A_ and EC_Exome-B_ (**Additional File 1: Table S33**; **Additional File 2: Figure S18**). We found a cosine similarity of 0.986 between EC_Exome-A_ and Fang et al. Signature A, while EC_Exome-B_ had a cosine similarity of 0.872 with Fang et al. Signature B.

Similar to what was observed in CRCs, dMutSα ECs were slightly younger than dMutLα, but this difference was not significant (median 60 *versus* 69 years; P=0.082; **Additional File 1:** **Table S28**). From this we again conclude that the increased CpG>TpG burden of dMutSα cancers is not a consequence of advanced age. While 17% of MMRd ECs harboured at least a mono-allelic *MBD4* truncation, only one was bi-allelic (in a dMutLα cancer), while no MMRd ECs displayed bi-allelic mutations in *TDG* or *UDG*. Like MMRd CRCs, there was no evidence of specifically increased CpG>TpG mutation or SBS1 in dMutLα (**Additional File 1:** **Table S34**) and dMutSα (**Additional File 1:** **Table S35**) ECs with somatic *MBD4* mutations.

While we were unable to map CpG>TpG mutations to their respective DNA methylation and replication timing (due to absence of tissue-specific reference data), we mapped C>T mutations in MMRd ECs to their respective transcription and replication strands. There was no transcription strand bias for CpG>TpG mutations in MMRp ECs, while non-CpG C>T mutations were more common on the template strand (**Additional File 1: Table S36**). In MMRd ECs, CpG>TpG mutations showed a coding strand bias, while non-CpG C>T mutations were biased to the template strand (**Additional File 1:** **Table S36**). Both CpG>TpG and non-CpG C>T mutations were biased to the template strand in *POLE-*mutant ECs, while there was no bias in *POLD1-*mutant, likely a reflection of the small number of cancers (**Additional File 1:** **Table S36**). We then performed the same analysis in dMutSα and dMutLα ECs (**Additional File 1: Table S37**; **Additional File 2: Figure S8B**). While CpG>TpG mutations showed a significant coding strand bias dMutLα ECs (median 1,727 *versus* 1,707; P=0.0012; Wilcoxon signed-rank test), no such bias was present in dMutSα cancers (median 6,257 *versus* 6,208; P>0.99; Wilcoxon signed-rank test). In contrast to this, non-CpG C>T mutations showed a template strand bias in both dMutLα (median 4,341 *versus* 3,985; P<2.2x10^-16^; Wilcoxon signed-rank test) and dMutSα (median 6,676 *versus* 6,089; P=0.0039; Wilcoxon signed-rank test) cancers. In line with what was observed in CRC, there was no difference in the log_2_(Coding/Template) ratio between dMutSα and dMutLα ECs for both CpG>TpG (median -0.0011 *versus* 0.016; P=0.25) and non-CpG C>T (median -0.131 *versus* -0.119; P=0.39) mutations (**Additional File 2: Figure S8B**), while the transcription strand bias was significantly greater for non-CpG C>T mutations than CpG>TpG in both dMutSα and dMutLα ECs (P=0.0039 and P<2.2x10^-16^ respectively; Wilcoxon signed-rank test; **Additional File 2: Figure S8B**).

As observed in CRC, MMRp ECs displayed a bias to the lagging replication strand for both CpG>TpG and non-CpG C>T mutations, while MMRd and *POLE-*mutant ECs displayed a significant leading strand bias for both types of C>T mutation (**Additional File 1: Table S38**). Furthermore, CpG>TpG mutations were more prevalent on the leading strand in dMutSα (median 4,366 *versus* 4,009; P=0.0039; Wilcoxon signed-rank test) and dMutLα (median 1,196 *versus* 1,095; P<2.2x10^-16^; Wilcoxon signed-rank test) ECs (**Additional File 1: Table S39**). Non-CpG C>T mutations also showed a leading strand bias in these dMutSα (median 5,561 *versus* 3,791; P=0.0039; Wilcoxon signed-rank test) and dMutLα (median 3,511 *versus* 2,727; P<2.2x10^-16^; Wilcoxon signed-rank test) cancers (**Additional File 1:** **Table S39**). There was no difference in the log_2_(Leading/Lagging) ratio of CpG>TpG mutations between dMutSα and dMutLα ECs (median 0.120 *versus* 0.149; P=0.58; **Figure 7B**), while this ratio was greater in dMutSα ECs than dMutLα for non-CpG C>T mutations (median 0.469 *versus* 0.362; P=0.024; **Figure 7B**). Consistent with CRCs, the leading strand bias of non-CpG C>T mutations was greater than that of CpG>TpG mutations in both dMutSα and dMutLα ECs (P=0.0039 and P<2.2x10^-16^ respectively; Wilcoxon signed-rank test; **Figure 7B**). The maximum CpG>TpG spectrum cosine similarity was reached between dMutSα and dMutLα ECs was achieved when 8,000 SBS1-associated mutations were added, approximately 56% of the total CpG>TpG burden. If these excess CpG>TpG mutations were deamination-associated, the expected log_2_(Leading/Lagging) ratio for dMutSα is 0.07. However, dMutSα ECs uniformly had a greater CpG>TpG log_2_(Leading/Lagging) ratio (P=0.0039, Wilcoxon signed-rank test). Therefore, consistent with the data from CRCs, it appears that at least some of the excess CpG>TpG mutations in dMutSα ECs are replication-associated.
